# Neural population geometry and optimal coding of tasks with shared latent structure

**DOI:** 10.1038/s41593-025-02183-y

**Published:** 2026-02-04

**Authors:** Albert J. Wakhloo, Will Slatton, SueYeon Chung

**Affiliations:** 1 Zuckerman Institute, Department of Neuroscience, Columbia University, New York, NY USA; 2 Center for Computational Neuroscience, Flatiron Institute, New York, NY USA; 3 Center for Neural Science, New York University, New York, NY USA; 4 Department of Physics and Kempner Institute for the Study of Natural and Artificial Intelligence, Harvard University, Cambridge, MA USA

**Keywords:** Neural decoding, Neural encoding, Sensory processing, Learning and memory

## Abstract

Animals can recognize latent structures in their environment and apply this information to efficiently navigate the world. Several works argue that the brain supports these abilities by forming neural representations from which behaviorally relevant variables can be read out across contexts and tasks. However, it is unclear which features of neural activity facilitate downstream readout. Here we analytically determine the geometric properties of neural activity that govern linear readout generalization on a set of tasks sharing a common latent structure. We show that four statistics summarizing the dimensionality, factorization and correlation structures of neural activity determine generalization. Early in learning, optimal neural representations are lower dimensional and exhibit higher correlations between single units and task variables than late in learning. We support these predictions through biological and artificial neural data analysis. Our results tie the linearly decodable information in neural population activity to its geometry.

## Abstract (web summary)

The authors analytically determine how neuronal correlations and geometry collectively determine readout generalization across tasks and show how these geometric features follow distinct trajectories over the course of learning.

## Main

Humans constantly solve different instances of similar problems. We brake at stop signs, stop at red lights and slow down in crowded streets. We do these things effortlessly and efficiently learn to use new sensory cues to regulate our behavior. This is possible because we are able to recognize overt symbols such as road signs as well as more abstract visual cues such as the crowdedness of a street. More generally, humans and other animals learn to recognize latent variables in their environment and use them to guide their behavior across contexts and tasks.

Recent experimental findings have described coding strategies that may underlie this ability. In particular, several studies have described cases where independent variables in the environment are represented along distinct directions of variation in the neuronal activity space^1–7^—for example, in orthogonal subspaces of the firing rates of a collection of neurons^2,7–9^. These independent factors have ranged from the contacts of distinct mouse whiskers^2^ to more abstract latent variables, such as the values of different choices in a decision-making task^3^. Neural representations in which distinct environmental variables are represented along independent or orthogonal directions of variation are referred to as factorized or disentangled. Factorized representations were recently shown to emerge in artificial networks trained on multiple tasks^8^ and are thought to support generalization to new contexts as well as efficient learning of new tasks that depend on shared latent variables^4,10^.

In a related line of work, studies have argued that the brain makes widespread use of cognitive maps to solve these problems^11^. These are coding strategies in which environmental variables are represented in the population code in a way that preserves task-relevant relations between them. This idea is supported by a range of findings—for example, studies in which structurally similar tuning profiles emerge in a neural population for distinct types of environmental variables^12–17^. These variables have ranged from an animal’s position to the frequency of an auditory stimulus^12^ to more abstract quantities, such as the amount of evidence accumulated in a decision-making task^14^. Here and in the findings referenced above, neural populations represent latent structure in the environment in a way that supports a target behavior. However, defining measures for and understanding why certain neural activity patterns represent latent variables in task-efficient ways remains challenging^18^.

A promising approach to tying neural activity patterns to computational goals is to study the geometry of neural responses^19^. Here, the overarching idea is to find which mesoscopic statistics of the population activity contribute to a macroscopic target computation or behavior. In this way, we can gain insight into neural computation without having to give a detailed account of microscopic single-unit activity. For example, in the domain of invariant object recognition, recent works analytically tied coding efficiency^20–23^ and few-shot generalization performance^24^ to measurable statistics of the population activity. In a related line of work, several studies tied the structure of neural correlations and population-level statistics to the information content of neural codes^25–29^. Recent studies have also investigated a wide range of motor^30,31^, sensory^2,6,15,32–35^ and decision-making^3,4,13,14^ computations and behaviors by analyzing the geometry of neural population responses.

In the present study, we develop an analytical theory for learning binary decision-making tasks depending on a common latent structure that directly ties the statistics of neural population responses to generalization performance. Although several authors have used linear probes or heuristic geometric measures to analyze population activity in such tasks (for example, refs. ^2,4^), to our knowledge there is no cohesive theory that directly ties mesoscopic statistical features of neural activity to task performance in these settings. To fill this gap, we analytically calculate how neural population geometry shapes the generalization error of an agent learning multiple tasks that depend on a common latent structure.

## Results

### Geometric measures govern generalization of linear readouts

**Fig. 1:**
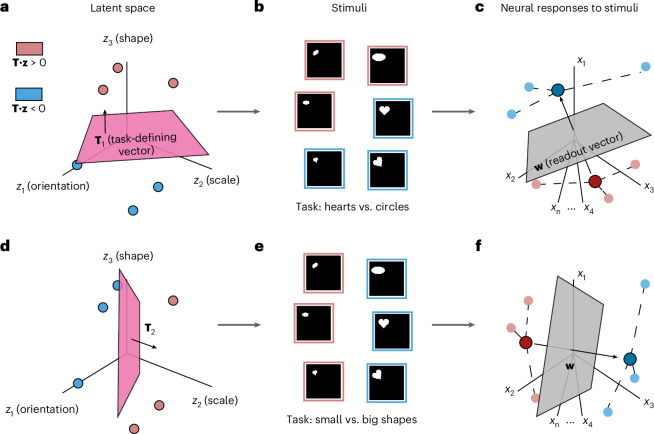
Schematic of the task and model setup using images from the dSprites dataset as an example. **a**,**b**, Stimuli in the dataset vary along a few latent dimensions, corresponding to the shape, orientation and position of the object. Thus, points in the latent space (**a**) can be directly mapped to visual stimuli (**b**). We form binary discrimination tasks, such as hearts versus circles, by linearly separating the latent space using a hyperplane with normal **T**_1_. **c**, Each stimulus elicits a neural activity pattern, visualized as points in an activity space. We form a linear readout of the neural activity by considering the difference between the mean activity pattern for circles (dark red) and hearts (dark blue). This readout corresponds to the activity of an idealized downstream unit with synaptic weights set by a supervised Hebbian learning rule (Methods)^40^. **d**,**e**, A new binary discrimination task (small versus big shapes) can be formed by separating the same set of stimuli using a different hyperplane with normal **T**_2_. **f**, For this task, the same supervised Hebbian rule leads to a new neural readout.

We study the ability of a neural population to support downstream learning of tasks that depend on a common latent structure. To do this, we start by considering a set of stimuli that vary along a few latent dimensions. For example, in the dSprites dataset depicted in Fig. 1a,b, different images can be described in terms of the shape, position and orientation of the shape in each image^36^. Within our modeling framework, this corresponds to the assumption that each stimulus can be mapped to a vector **z** in an underlying *d*-dimensional latent space. In response to each stimulus, we consider a vector **x** of neural responses—for example, the firing rates of a population of neurons to each image in the dataset (Fig. 1c). Thus, our modeling framework considers datasets comprising latent vectors describing individual stimuli, together with neural activity patterns associated with each stimulus.

Within this setting, we determine how well neural responses can be used to solve binary tasks involving the latent variables. The task labels for a given binary classification task are formed by linearly separating the latent space into two pieces using a hyperplane with normal vector **T**^37–40^. In the example shown in Fig. 1, different choices of **T** generate shape categorization (Fig. 1a,b) or size categorization (Fig. 1d,e) tasks. Note that, although the separation is linear in the latent space, it may be highly nonlinear in the stimulus space.

We quantify how well these neural activity patterns support downstream classification by considering the performance of a simple linear readout mechanism. Specifically, we calculate the generalization error of an idealized downstream unit that receives inputs from all neurons in the population and adjusts its synaptic weights according to a supervised Hebbian plasticity rule (Methods). The activity of this downstream unit is then determined by a weighted summation of its inputs, followed by a threshold operation. This unit’s activity acts as a difference of means classifier when the number of labels for each class is equal (Fig. 1c–f)^40^. We calculate the generalization error of this readout after being trained on a dataset of *p*-many stimuli both for a single, fixed task (for example, the hearts versus circles task in Fig. 1b) as well as the average generalization error across all possible tasks. In this way, we connect the statistical properties of the neural code to the multitask learning problem.

**Fig. 2:**
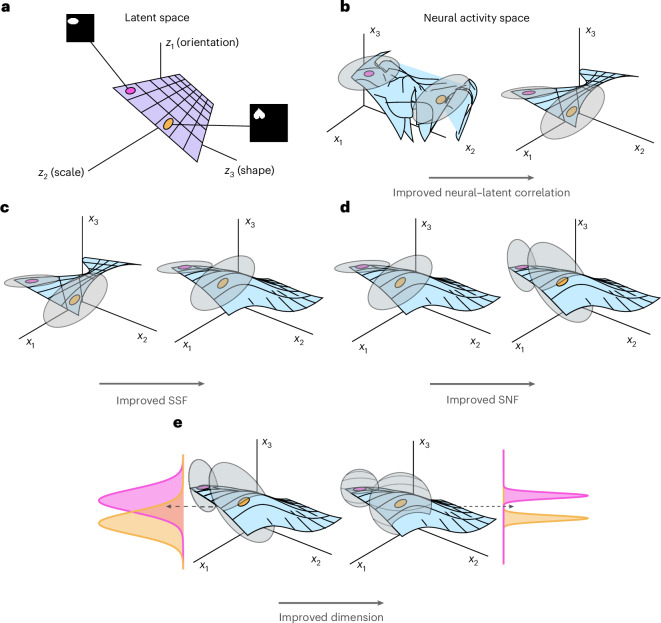
Schematic of the geometric terms. We visualize different possible neuronal activity patterns elicited by the same set of stimuli. **a**, A small slice of the latent space from which stimuli are generated. **b**, Visualization of neural activity patterns responding to this set of stimuli with low (left) and high (right) total correlation. When the correlation is high, the relative distances between points in the latent space are approximately preserved in the neural state space. **c**, SSF. When the SSF is low, different latent variables are represented along overlapping directions; when it is high, independent latent variables are represented along uncorrelated directions of variability in the neural state space. **d**, SNF. When the SNF is low, the noise distribution (gray ellipses) around a point in the firing rate space falls along the coding directions; when it is high, the noise distribution is uncorrelated with these directions. **e**, Neural dimension. In higher-dimensional representations, the neural activity and associated noise distribution occupy more directions in the state space, shown here as two-dimensional (left) versus three-dimensional (right) noise distributions. As the dimension increases, the projection of a sample of neural activity onto a given direction becomes increasingly concentrated, supporting generalization performance^24^.

Using this simplified model, we derive a formula for the average generalization error. As described in the Supplementary Information, we prove that, under a certain Gaussian approximation, the error *E*_*g*_ is given by

1 $${E}_{g}=\frac{1}{\pi }{\tan }^{-1}\left(\sqrt{\frac{\pi }{2p{c}^{2}{\rm{PR}}(\mathbf{\Psi} )}+\frac{1}{f}+\frac{1}{s}-1}\right),$$

where we have written the generalization error as a strictly decreasing function of four geometric terms as well as the number of samples in the training dataset, *p*. In Fig. 2, we visualize different geometries that could arise in response to a fixed set of stimuli (Fig. 2a). We now discuss each of these terms one by one (see Methods for precise definitions and Supplementary Information section 3 for details):

- **Neural–latent correlation:** *c*. This term is a normalized sum of squared covariances between neurons and latent variables. As such, it measures the overall correlation between single-unit responses and latent variables. At the population level, the neural–latent correlation measures how sensitive the population activity is to variations in the latent space (Fig. 2b).
- **Signal–signal factorization (SSF):** *f*. This term measures the alignment between the coding directions of distinct latent variables. The SSF term favors neural coding schemes that represent independent latent variables along uncorrelated directions in the neural state space. Moreover, this term encourages these representations to devote equal variance to each independent latent factor—that is, to form a whitened representation of the latents (Fig. 2c).
- **Signal–noise factorization (SNF):** *s*. This term measures the magnitude of the noise that lies along the coding directions of the latent variables. Ideally, any noise present in the neural responses should lie in directions that are uncorrelated with the directions representing the latent variables^25,27^ (Fig. 2d).
- **Neural dimension:** ${\mathrm{PR}}({\mathbf{\Psi}})$. The participation ratio of the neural responses measures the effective number of dimensions that the population activity spans. When all else is equal, higher-dimensional responses are preferred, as neuronal noise is less correlated from trial to trial^24^ (Fig. 2e).

Finally, we note that the contribution to the error of the neural dimension and neural–latent correlation decays to zero with the number of training samples (Methods). On the other hand, the error stemming from the SSF and SNF represents an ‘irreducible error’ that does not depend on the number of training samples. Intuitively, in the few-shot regime, the main concern is maximizing the amount of total signal in the neural code while minimizing the impact of noise. These two aspects are primarily controlled by the total correlation and dimension, respectively. On the other hand, in the many-shot regime, the main concern becomes keeping the representations of distinct latent variables separate from one another and separate from noise directions. The two factorization terms control these features of the code. These considerations suggest that certain geometries, which perform well early in learning, when *p* is small, may be suboptimal late in learning. We make these intuitions precise in ‘Optimal representation of latent variables’ below.

### Geometry of multitask learning in multilayer perceptrons

**Fig. 3:**
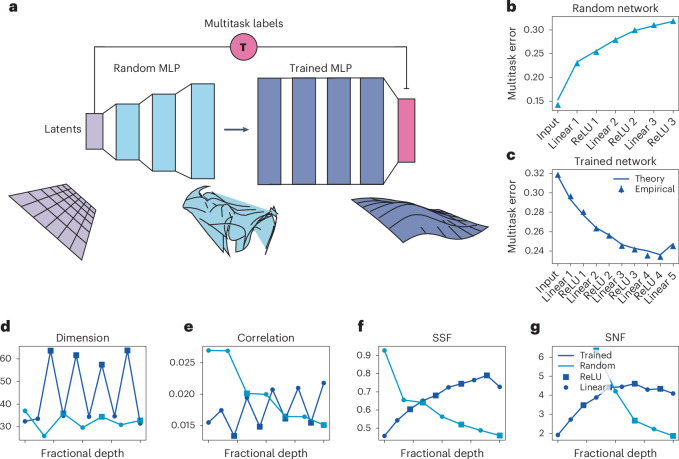
Theory predicts generalization error of the Hebbian rule in trained and random MLPs. **a**, Schematic of the simulation. Latent variables **z** are randomly shattered to generate task labels. These latents are passed through a random MLP (light blue) and are then used as inputs to train a three-hidden-layer MLP (dark blue) on the multitask binary classification problem using stochastic gradient descent. **b**,**c**, After training, we sample a new set of latents and teacher vectors and calculate the generalization error of the Hebbian rule on each layer of the random (**b**) and trained (**c**) network. Theoretical predictions closely track empirical errors, and the trained network achieves a lower error in later layers. **d**–**g**, Geometric terms across layers for the random (light blue line) and trained (dark blue line) networks. Linear layers are marked by circles and ReLU layers by squares. Interestingly, the error only slightly changes across linear and ReLU layers of the same model stage, in spite of sharp changes in the geometry. In the trained network, the application of ReLU consistently causes increases in the dimension and SSF as well as decreases in the correlation.

In practice, neural data are complex and may be non-Gaussian. To test our theory in this regime, we now apply our formula for the generalization error (equation (1)) and its geometric decomposition to both random and trained nonlinear multilayer perceptrons (MLPs). As shown in Fig. 3a, we first sample a set of zero mean Gaussian latent variables **z**. We then feed these latent variables through a random MLP with increasing hidden layer sizes. After generating this set of stimuli, we sample 500 random **T** vectors and use these to generate a set of task labels (Methods). These labels and data points from the random MLP are then used to train a downstream three-hidden-layer MLP, which predicts the label for each task using a task-specific linear readout from the shared penultimate layer. This is a multilayer version of the hidden manifold modeling framework from ref. ^38^. Finally, we sample a new set of latent variables along with a new set of tasks and calculate the generalization error of the Hebbian rule when applied to the representations at different layers of the trained and untrained MLPs (Methods). This setup allows us to validate our theory on nonlinear transformations of Gaussian latent variables.

As shown in Fig. 3b,c, we find good agreement between our formula, equation (1), and the empirical generalization error. Turning to our geometric decomposition of the error, we find several interesting trends across layers and nonlinearities (Fig. 3d–g). Most notably, in the trained network, we find that linear and rectified linear unit (ReLU) layers orchestrate a tradeoff between the geometric terms that together lead to an overall decrease in multitask generalization error. As shown in Fig. 3d–g, the neural dimension spikes each time the nonlinearity is applied, at the cost of the total correlation. Conversely, at each linear layer, the correlation sharply rises while the dimension falls. On the other hand, we find that both the SSF and SNF terms monotonically increase through the penultimate layer of the network. Notably, this pattern is not present in the random network. Thus, the trained MLP learns to use the nonlinearity to increase the dimension of the representation as well as the factorization of the latent variables while simultaneously squashing particularly harmful noisy directions of variability in the input. We visualize the training dynamics that lead to these representations in Supplementary Fig. 4. Although these are sharp changes in the geometry, the overall generalization error across layers exhibits only minor fluctuations between most linear and ReLU layers, highlighting the limitations of a generalization error-based approach to analyzing network activity without studying the underlying geometry.

### Disentangling in deep pose estimation networks

**Fig. 4:**
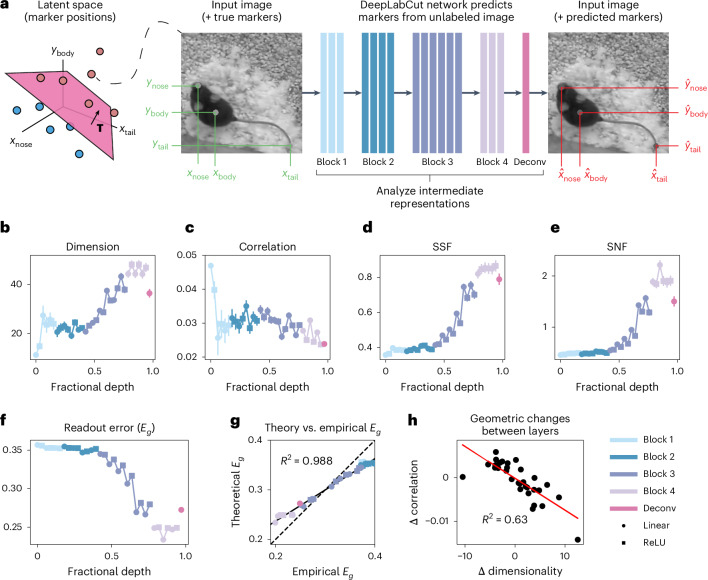
Disentangling of animal pose parameters by a deep neural network. Data are reported as means ± s.e.m., where statistics are over 20 random projections of the network representations down to a fixed dimensionality of *n* = 100. **a**, A DCNN trained with the DeepLabCut framework^41^ predicts the position of 12 markers (only three are illustrated here) in unlabeled images of an adult mouse (*N* = 1) (Methods and ref. ^42^). We treat the *d* = 24 total (*x*, *y*) coordinates of these marker positions as latent variables and study how the DCNN disentangles them from the image inputs. **b**, The total dimensionality of the DCNN’s intermediate representations increases across layers. **c**, The total correlation between the latents and the network’s internal representations decays across layers, representing a tradeoff with dimensionality. **d**,**e**, Both SSF and SNF improve monotonically across layers. **f**, Multitask error decreases across layers, indicating successful and gradual disentangling of the latent variables across layers of the DCNN. Here, *p* = 150 training samples are used, and we calculate the error using equation (1). **g**, Our theoretical expression for multitask error in terms of our four geometric terms is not quantitatively exact for this complex natural dataset but, nonetheless, captures essentially all variance in the true multitask error. **h**, For each pair of adjacent layers in the network, we plot the change in the neural–latent correlation and the dimensionality term.

To test the validity of our theory on more complex natural data, we study the representations of animal pose in a deep convolutional neural network (DCNN) trained to estimate pose parameters from natural images (Fig. 4a)^41,42^. In this setup, the *d* = 24 dimensional latent variables $\mathbf{z}\in {{\mathbb{R}}}^{24}$ consist of (*x*, *y*) coordinates for 12 different ‘marker’ locations on a mouse’s body (for example, the left paw or the tip of the tail). We calculate the average multitask error generated by randomly shattering this latent space (Fig. 4a) both empirically and using our formula, equation (1). The distribution of the latent variables is highly non-Gaussian and provides a challenge for our theory. The pose estimation network consists of a fine-tuned ResNet50 and a final trained deconvolutional layer with 12 output channels, one for each marker location. This can be viewed as a multitask architecture in which the output of the fine-tuned ResNet50 produces a shared representation that is read out by 12 different deconvolution kernels to estimate the position of each independent marker.

The task-averaged generalization error *E*_*g*_ improves across the layers of the network (Fig. 4f). Interestingly, the geometric changes driving this improvement in *E*_*g*_ are qualitatively different than in the random MLP case. We observe a similar tradeoff between the dimension and correlation terms at each layer (Fig. 4h, compare to Fig. 3d,e), but this network prioritizes expanding the data dimensionality (Fig. 4b) while sacrificing correlation (Fig. 4c). In this more challenging setting with highly non-Gaussian latents and complex natural inputs, our analytical expression for *E*_*g*_ is slightly biased (Fig. 4g). However, the analytical prediction for *E*_*g*_ still linearly explains practically all of the variance in the empirically computed multitask error (*R*^2^ = 0.988). Thus, our geometric terms capture the representational changes that drive the observed trends in *E*_*g*_.

### Predicting readout performance of macaque visual representations

**Fig. 5:**
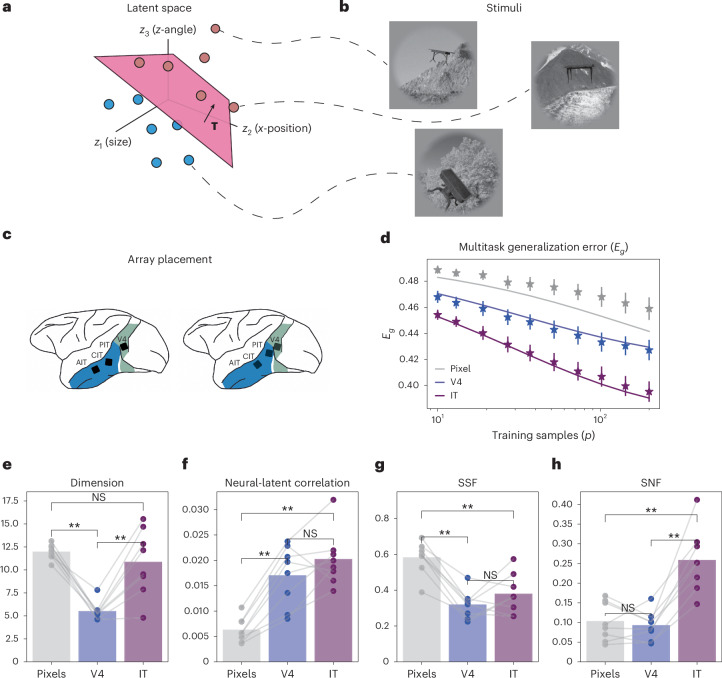
Theory predicts multitask error in macaque V4 and IT data. **a**,**b**, Example stimuli and tasks^43^. Visual stimuli included 64 objects grouped into eight categories and were generated by modifying *d* = 6 continuous latent variables that included object size, position and angle. We form binary classification tasks on subsets of the data coming from the same category—for example, all images from the ‘Tables’ category. **c**, Array placement. Figure was adapted with permission from ref. ^43^. **d**, Generalization error across pixels and brain regions calculated empirically (markers) and using our formula for the generalization error (solid line). Error bars denote the standard error across categories. **e**–**h**, Geometric terms across pixels and neural responses for each category. Bars denote the mean, and asterisks denote a significant difference (*P* < 0.01, two-sided paired *t*-test, *N* = 8 object categories; Supplementary Table 1). NS, not significant.

Having considered nonlinear artificial neural networks, we now apply our theory to biological neural data. To do this, we draw from preexisting multi-unit recordings from macaque V4 and inferior temporal cortex (IT) taken while two monkeys viewed visual stimuli (Methods and Fig. 5a–c)^43^. The stimuli used in these experiments included images of 64 objects taken from eight categories and were generated by modifying *d* = 6 continuous latent variables that included the size, position and angle of the object. This allows us to form binary classification task labels by shattering this six-dimensional latent space on subsets of the data corresponding to individual object categories; for example, a given task could involve separating images of chairs on the left side of the screen from those on the right. We test the validity of our generalization error equation, equation (1), by calculating the error of Hebbian readouts applied to V4 and IT neural responses as well as the raw pixel values. We find that our formula accurately predicts the generalization error, particularly for the neural response readouts (Fig. 5d). Furthermore, we find that generalization error improves through the ventral stream, in line with previous results^44^.

From equation (1), we can see that there are many different geometries that yield the same generalization error. Thus, it is a priori unclear how these measures ought to change from the pixel space through the ventral stream. Applying our geometric decomposition of the generalization error to these data, we find that the dimension is lower in V4 than in either IT or the pixel data^24^ (Fig. 5e) and that the correlation increases from the pixels to the visual areas. The transformation from pixels to V4 is reminiscent of the tension we found between correlation and dimension in the preceding sections, whereby transformations that improve generalization error by increasing neural–latent correlation can sometimes do so at the price of a slightly lower dimension (and vice versa). Turning to the factorization measures, the most dramatic trend is the increase in SNF in IT. This suggests that, in IT, latent-unrelated variability overlaps less with the coding directions than in V4 or the pixels (Fig. 5e–h). Taken together, these results show that our formula for the generalization error (equation (1)) captures empirical readout performance and demonstrates the applicability of our metrics as a tool for tying the geometry of neuronal population responses to the computational objective of multitask learning.

### Optimal representation of latent variables

**Fig. 6:**
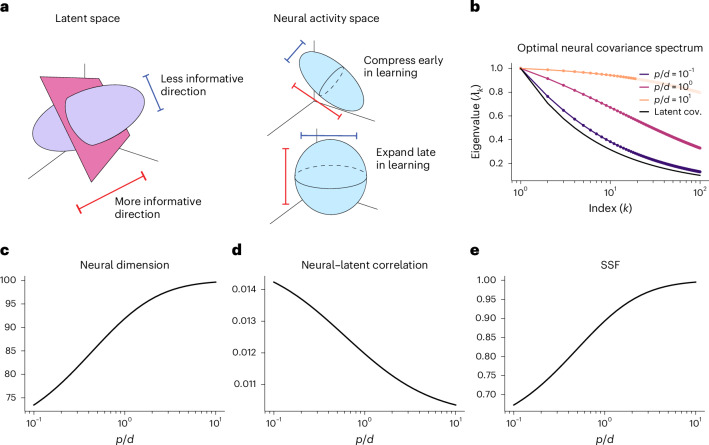
Optimal representational geometry over learning. **a**, In our task setup, directions in the latent space that have little variance are, on average, less informative of the task labels (Methods and Supplementary Information section 4). **b**, Eigenvalues of the optimal neural covariance as a function of the number of samples per latent dimension, *p* / *d*. We show the eigenvalues of the latent variables’ covariance in black. Markers correspond to results obtained by optimizing our formula for the generalization error numerically, and solid lines correspond to our formula for the optimal code’s spectrum (Methods). As training proceeds (that is, as *p* increases), the spectrum of the optimal neural code becomes increasingly flat. **c**–**e**, Optimal representational geometry at different stages of training. Early in training, optimal representations have a higher neural–latent correlation and a lower dimension and SSF than they do late in training. (Note that we do not plot the SNF, as optimal representations in our framework are noiseless or have noise that is uncorrelated with the signal directions. In this regime, the SNF diverges for all *p*).

Our theory provides an ideal framework to pose normative questions regarding multitask learning. In our model, latent variables that have less variance contain, on average, less information about the task labels (Fig. 6a and Supplementary Information section 4). For the data analyses presented in the previous section, we normalize the latent variables so that they are all weighted equally. However, this aspect of our framework allows us to study how the structure of the latent variables and the number of available training samples determine which neural representations are optimal.

We show analytically that the optimal representation disentangles latent variables (Supplementary Information section 4). More precisely, we show that when the latent variables are not correlated with one another, neural responses represent distinct latent variables along orthogonal directions. When the latent variables are correlated with one another, we show that the principal components of the latent variables directly map onto the (mutually orthogonal) principal components of the neural activity (Supplementary Information section 4). Thus, disentangled representations are optimal in this framework.

How does the structure of the latent variables and the degree of learning affect the geometry of optimal neural representations? In addition to being disentangled, we find that the optimal code compresses less informative variables early in learning (that is, when *p* is small) and expands these variables late in learning. For an optimal code, independent latent variables are represented along uncorrelated directions; however, the variance along each direction is not equal. As shown in Fig. 6a, early in training, less informative latent variables correspond to directions in the neuronal activity space that have small variance. As learning proceeds (that is, as *p* increases), the amount of variance in neural space dedicated to these less informative latent variables grows (Methods and Supplementary Information section 4). Intuitively, the optimal code only starts paying attention to the less informative latent variables when there are enough samples to learn their relevance to a given task.

We trace these features of the optimal neural code back to the eigenvalues of the neuronal covariance matrix and our geometric terms. As shown in Fig. 6b, the eigenspectrum of the optimal code becomes increasingly flat over the course of learning. This reflects the fact that more and more variance is being dedicated to the less informative directions in the latent space (that is, directions with smaller variance). Arranging the eigenvalues of the latent variables’ covariance matrix in descending order, *ω*_1_ ≥*ω*_2_ ≥ … ≥ *ω*_*d*_, we show that the optimal code’s covariance matrix has at least *d* non-zero eigenvalues that are given by:

2 $${\psi }_{i}=C\frac{{\omega }_{i}}{2p{\omega }_{i}+\pi {\sum }_{k}{\omega }_{k}},$$

where *C* is an arbitrary constant. We can see that, as *p* grows, the spectrum becomes increasingly flat, reflecting the expansion strategy (Methods and Supplementary Information section 4).

Turning to our geometric measures, we find that, early in training, optimal neural codes are lower dimensional with higher neural–latent correlations than they are late in training (Fig. 6c–e). A key normative prediction from our theory is, therefore, that the task-related variability—that is, the signal variance—of neural responses becomes increasingly high dimensional as an agent learns to perform tasks that depend on a complex latent structure. Moreover, these results suggest that the strength of the correlations between single units and task variables may decrease in the late stages of learning. We present evidence for these predictions in the following section.

### Geometry of spatial representations in prefrontal cortex and CA1 during learning

**Fig. 7:**
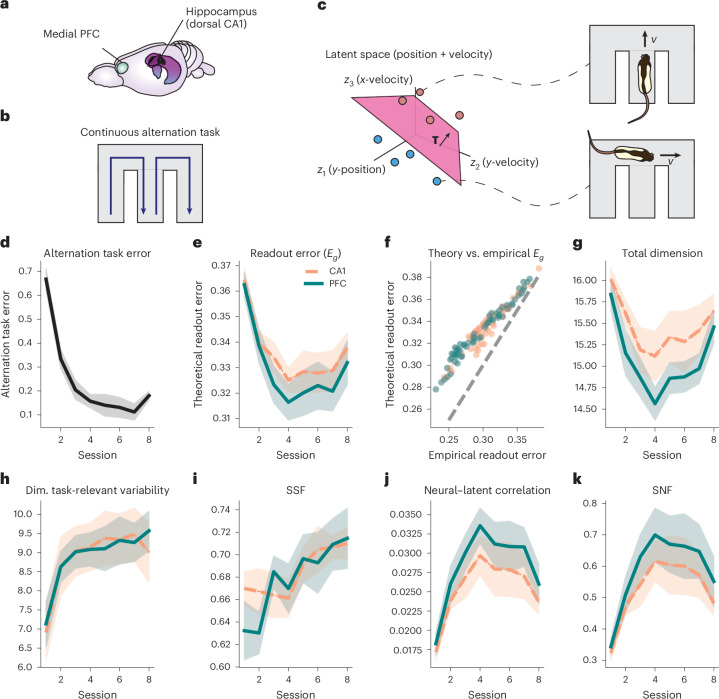
Changes in the geometry of spatial representations during learning in PFC (solid) and CA1 (dashed). **a**, Multi-unit recordings were obtained from medial PFC and dorsal CA1 (ref. ^45^). Figure is adapted from ref. ^45^. **b**, Rats performed a continuous alternation task in a W-maze. **c**, For our multitask readout analysis, we treated the *x* and *y* positions and velocity values as latent variables from which binary classification tasks were formed. **d**,**e**, Behavioral error on the continuous alternation task and neural readout error quickly decrease over the first few sessions, leading to a large correlation between the behavioral and readout errors on single sessions (*R*^2^ = 0.49 (CA1) and *R*^2^ = 0.51 (PFC); Supplementary Fig. 12c). **f**, Empirical generalization error versus our estimate from equation (1). Although there is a small bias for low error values, we find a good agreement overall. **g**–**k**, Geometric terms over the course of learning. Lines denote the mean within a region, and error bars denote the standard error (*N* = 8 rats).

We now analyze the geometry of navigational representations in prefrontal cortex (PFC) and CA1 as rats learn to perform a continuous alternation task over the course of eight sessions (Fig. 7a,b)^45^. To do this, we analyze the degree to which an animal’s position and velocity can be decoded from population firing rates. Specifically, we treat the animal’s *x* and *y* position and velocity as latent variables from which we form binary classification tasks (Fig. 7c). We then calculate the task-averaged readout error, *E*_*g*_, and geometric measures from PFC and CA1 firing rate data for each session (*n* ≥ 19 units; Methods). The session-by-session neural readout errors *E*_*g*_ thus quantify the ability of a linear readout to decode position and velocity information through the course of learning. These readout errors decrease over learning (Fig. 7e,f) and are correlated with single-session behavioral task performance (*R*^2^ = 0.48 (CA1) and *R*^2^ = 0.51 (PFC); Supplementary Fig. 12c).

We now analyze the trends in the readout error and geometry during learning. Over the first four sessions, both behavioral and neural readout error rapidly improve (Fig. 7d,e). During this time, nearly every geometric measure increases (Fig. 7h–k). Interestingly, we find that although the total neural dimension drops over these initial sessions (Fig. 7g), the dimension of the subspace containing task-relevant variability increases (Fig. 7h) (Methods). This suggests that the initial drop in the dimension is driven by a compression of navigation-irrelevant activity. After session four, both behavioral and readout error largely plateau. At this point, several of these initial trends reverse; in particular, the total dimension begins to increase, whereas the correlation drops.

To test the significance of these non-monotonic effects of learning on the geometry, we fit mixed-effects quadratic regression models for each geometric term (Methods and Supplementary Fig. 12). We found strong, significant effects for both the linear and quadratic terms in PFC and CA1 for the total dimension, correlation and SNF (*P* < 10^−5^; Supplementary Table 2). In each case, the sign of the quadratic term was the opposite of the linear term, demonstrating the non-monotonic effects of learning on these quantities. We did not find evidence for a non-monotonic trend in the SSF in either region (*P* > 0.05). Although we did find weaker significant effects of the quadratic model for the task-relevant dimension (*P* < 0.01), the model was a comparatively poorer fit to this quantity in PFC (Supplementary Fig. 12). As such, we carried out a follow-up analysis using mixed-effect linear models, which demonstrated significant, monotonic effects of learning on the SSF and task-relevant dimension across both regions (*P* < 10^−3^ SSF and *P* < 10^−5^ task dimension; Supplementary Table 2).

How do these trends in the geometry align with our normative predictions? In the previous section, we analyzed the behavior of optimal representations over the course of learning. However, the initial state of the network is likely far from any kind of optimality, as suggested by the very large drops in behavioral error (Fig. 7d), readout error (Fig. 7e) and increases in nearly every geometric measure (Fig. 7h–k) over the first four sessions. (Indeed, we find similar geometric trends for the training dynamics of the multi-task MLPs around initialization (Supplementary Fig. 4).) Once the readout and behavioral errors begin to plateau at session four, we find that the ensuing trends are consistent with our predictions for optimal representations: the dimensionality measures and SSF increase over learning, whereas the neural–latent correlation decreases. In other words, once the network has formed a representation from which task variables can be efficiently decoded, the trends in the geometry align with the behavior we found for optimal representations over learning.

## Discussion

In this work, we analyzed a model of learning multiple tasks that share a common latent structure. We showed that the generalization error in this model decomposes into four terms summarizing the correlation structures, factorization and dimension of the neural activity. Our work complements previous studies of multitask learning^46–48^ and neural correlations^25–27,29,49–52^ by analytically describing how noise correlation structures interact with signal correlations, dimensionality and signal factorization to collectively determine readout generalization performance.

We leveraged these analytical results to determine which geometries minimize the generalization error, given a fixed latent structure and number of available training samples. In addition to being disentangled, we found that the task-relevant subspace of optimal neural codes shifts from being low to high dimensional as the number of available samples increases. This reflects a strategy of compressing less useful information when data are scarce and expanding it when data are abundant. Note that, in a given dataset, low-variance latent variables may be of greater interest than those with high variance. To apply our theory to such situations, one can bias the linear shatterings to emphasize low-variance latent variables. As discussed at the end of Supplementary Information section 1, this is equivalent to rescaling individual latent variables.

It is interesting to compare these findings to previous work reporting that higher-dimensional representations of individual object classes are preferred for invariant object recognition in the few-shot learning setting^24^. Although the contribution to the error of the neural dimension scales as 1 / *p* in our formula just as in this work, the additional contribution to the error of the SSF and SNF terms, together with the constraint of the covariance matrix remaining positive semi-definite, leads to optimal representations with lower dimension in the few-shot regime. These results highlight the fact that different geometric terms may compete with each other in ways that cannot be directly read off from generalization error equations when there are additional constraints imposed on the system.

These optimal coding trends may hold for other forms of readout, beyond the Hebbian learning rule considered here. In particular, theories of linear least squares estimation suggest that similar phenomena may occur for regression tasks with more complex readouts^53–56^. Furthermore, we compared our theoretically calculated Hebbian readout error to the empirical generalization error of a linear support vector classifier (SVC) trained on the same tasks. Across every analysis reported in this paper, we found a strong correlation between SVC and Hebbian errors, particularly in the brain data, suggesting that the geometric terms described here are also informative of the generalization errors of other linear readouts (Supplementary Fig. 3). A related line of work has considered the role of neural dimension in random pattern separation^57–59^. In these settings, the optimal dimension can decrease when neural noise increases, and future work can examine whether a similar relation holds in the multitask learning setting considered here.

To test our theory, we first carried out a series of analyses on artificial networks. Across both networks, we found a tension between the neural dimension and correlation: layers that yielded a sharp increase in the dimension typically did so at the cost of the correlation and vice versa. These results illustrate a tradeoff between a population code’s dimensionality and the amount of correlated variability between single units and task variables. In light of previous work demonstrating the interaction between the baseline firing rate of a population and neuronal correlations, it would be interesting to analyze how population sparsity and single-unit response reliability interact with the neural dimension and our neural–latent correlation statistic in recordings or models of tuned sensory neurons^28,29^.

We next applied our theory to electrophysiological recordings from macaque V4 and IT^43^ as well as navigational representations in rat CA1 and PFC^45^. Both of these analyses were carried out using multi-unit recordings, and, thus, these trends should be interpreted with caution. Geometric measures calculated from these recordings, such as the neural dimension, may not necessarily be indicative of the dimension of the entire population within a region. For example, the local similarity of tuning properties within certain visual regions may artificially decrease dimension, and systematic differences in recording quality between regions could artificially inflate the SNF estimates. Although these caveats should be kept in mind, we found a sharp increase in the SNF from the pixels and V4 to IT, suggesting that latent-unrelated variability may become increasingly orthogonal to the coding directions through the ventral stream. Indeed, orthogonalization of neural variability was previously observed in early visual areas, and it would be interesting to study the evolution of this phenomenon across the cortical hierarchy^25,34^.

Turning to the rat CA1 and PFC analyses, we found that the SSF and the dimension of the subspace containing task-relevant navigational information increase over the course of learning. Furthermore, we found that the correlation first increases and then decreases. These trends are consistent with the predictions that we derived by considering optimal neural representations. Together, these results demonstrate the applicability of our theory to probing neural representations across brain regions and through learning.

In this work, we analyzed linear readout applied to tasks that came from shattering a continuous latent space. Determining which geometric measures are relevant for common nonlinear decoders remains an important open problem. Furthermore, our modeling framework makes several distributional assumptions, and future work could extend this theory to consider distributions of latents and tasks that more closely mirror common experimental settings. One could repeat our general calculation while restricting the distribution of task vectors to a handful of relevant directions or restricting the distribution of latent variables to be fixed to discrete values. This would be particularly interesting in settings where only particular groups of tasks or latent variable values are relevant for an animal to consider. As a further extension, it would be interesting to extend this work to consider the generalization error on a test set with distinct statistical properties. We plan to pursue these lines of research in subsequent work.

## Methods

### Model of multitask learning

We model a setting in which an agent learns a set of binary classification tasks by performing linear readout on a set of neural activity vectors. Formally, we assume that each stimulus is associated with a *d*-dimensional latent vector ${\mathbf{z}}_{\mu }\in {{\mathbb{R}}}^{d}$, with 1 ≤ *μ* ≤ *p* denoting the sample index. The labels for a specific task are formed by shattering the latent space using a hyperplane with a normal vector, **T**. Thus, the binary classification task labels, *y*_*μ*_, satisfy the relation, *y*_*μ*_ = sign(**T·z**_*μ*_), so that each **T** vector defines a specific classification task. Associated with each latent, we also consider *n*-dimensional neural activity patterns, ${\mathbf{x}}_{\mu }\in {{\mathbb{R}}}^{n}$. From these data, the agent then forms predictions of new data points using a supervised Hebbian readout rule^40^. More precisely, given a new stimulus associated with latent variables **z**_+_ and firing rates **x**_+_, the agent forms a prediction ${\hat{y}}_{+}$ using the rule

3 $${\hat{y}}_{+}={\rm{sign}}(\mathbf{w}\cdot {\mathbf{x}}_{+}),\quad \mathbf{w}=\frac{1}{p}\sum _{\mu }{y}_{\mu }{\mathbf{x}}_{\mu }.$$

When the labels are balanced, this corresponds to using the difference in the mean activities for positively and negatively labeled examples (Fig. 1f; see Supplementary Fig. 3 for results on SVC classifiers). We evaluate how well the neural code can support downstream classification by calculating the generalization error of these predictions, averaged across different tasks (that is, different **T** vectors), although we also calculate the error for a fixed **T** along the way (Supplementary Information section 1).

Although the generalization error for arbitrary distributions of neuronal activities and latent variables is analytically intractable, we show that, in many cases, the error only depends on a few key statistics. To do this, we draw from work in deep learning theory describing a Gaussian equivalence principle (GEP), which states that the generalization error of linear readouts trained on complex distributions can, in many cases, be well approximated by studying simpler Gaussian models^38,39,60,61^. We analytically calculate the generalization error using a Gaussian model and show empirically that our theory accurately predicts the generalization error of the linear readout rule when applied to more complex settings. Formally, we assume that each pair of neural responses and latent variables (**x**_*μ*_, **z**_*μ*_) are jointly zero mean Gaussians with covariance matrices:

4 $${\mathbb{E}}[\mathbf{xx}^{\top }]=\mathbf{\Psi} ,\quad {\mathbb{E}}[\mathbf{xz}^{\top }]=\mathbf{\Phi} ,\quad {\mathbb{E}}[\mathbf{zz}^{\top }]=\mathbf{\Omega} .$$

We can see that **Ψ** describes the neuron–neuron covariances; **Φ** contains the covariances between single-unit responses and the latent variables; and **Ω** describes covariances between latent variables in the dataset. This model corresponds to a variant of the popular student–teacher model^37,39^. Insofar as the GEP holds, the neuronal and latent (cross-)covariances fully specify the generalization error of the linear readout.

In our analyses below, we enforce the zero mean condition by centering the **x** and **z** data matrices. Note that because the **T** vectors are chosen randomly from a Gaussian distribution, the latent covariance **Ω** determines which directions in the latent space are, on average, most informative of the task labels. Directions in the latent space that have a significant amount of variance are, on average, more informative of the task labels in this setup (Supplementary Information section 4; note that one can simply whiten latent variables when applying this framework to data to circumvent this feature).

### Decomposition of generalization error across tasks

We prove a formula for the generalization error of the linear readout, averaged across different binary classification tasks (that is, different **T** vectors). To do so, we consider the limiting case in which the number of neurons, latent dimensions and training samples are all large and of similar size. Furthermore, we assume that the covariance matrices given above satisfy certain spectral properties (Supplementary Information section 1). Under these assumptions, we show that the average generalization error, *E*_*g*_, given a training dataset of size *p*, is a decreasing function of the four geometric terms. Formally, we have

5 $${E}_{g}=\frac{1}{\pi }{\tan }^{-1}\left(\sqrt{\frac{\pi }{2p{c}^{2}{\rm{PR}}(\mathbf{\Psi} )}+\frac{1}{f}+\frac{1}{s}-1}\right),$$

where we have introduced the total neural–latent correlation *c*, the SSF *f*, the SNF *s* and the dimension of the population responses as measured by the participation ratio, PR(**Ψ**). These terms correspond to statistics of the covariance matrices specified above:

6 $$c=\frac{{\mathrm{Tr}}({\mathbf{\Phi}}{\mathbf{\Phi}}^{\top })}{{\mathrm{Tr}}({\mathbf{\Psi}}){\mathrm{Tr}}({\mathbf{\Omega}} )}$$

7 $${\rm{PR}}(\mathbf{\Psi} )=\frac{{\rm{Tr}}{(\mathbf{\Psi} )}^{2}}{{\rm{Tr}}({\mathbf{\Psi} }^{2})}$$

8 $$f=\frac{{\rm{Tr}}{({\mathbf{\Phi}}{\mathbf{\Phi }}^{\top })}^{2}}{{\rm{Tr}}(\mathbf{\Omega} ){\rm{Tr}}(\mathbf{\Phi }^{\top }\mathbf{\Phi} {\mathbf{\Omega} }^{-1}{\mathbf{\Phi} }^{\top }\mathbf{\Phi} )}$$

9 $$s=\frac{{\mathrm{Tr}}{({\mathbf{\Phi}}{\mathbf{\Phi }}^{\top })}^{2}}{{\mathrm{Tr}}(\mathbf{\Omega} ){\mathrm{Tr}}({\mathbf{\Phi} }^{\top }(\mathbf{\Psi} -\mathbf{\Phi} {\mathbf{\Omega} }^{-1}{\mathbf{\Phi} }^{\top })\mathbf{\Phi} )}$$

Because the function $F(w)=\frac{1}{\pi }{\tan }^{-1}\left(\sqrt{w-1}\right)$ is strictly increasing, we can see that the error is a decreasing function of each of these geometric terms. Note that, in writing equation (5), we have separated a term that depends on the number of training samples, 1 / [*c*^2^PR(**Ψ**)], from a term that is independent of the sample size, 1 / *f* + 1 / *s*. Thus, we can see that the correlation and dimension terms become less important as *p* grows.

We give a detailed discussion motivating the mathematical definitions of these terms in Supplementary Information section 3. To summarize, the definition of *c* corresponds to a normalized sum of squared covariances between all single units and latent variables and is, thus, a multidimensional generalization of the Pearson correlation coefficient between neural responses and latent variables. The participation ratio is a standard measure of the dimension of a neural representation (see, for example, refs. ^57,58^). Turning to the factorization measures, the term *f* measures the overall degree to which independent latent variables are represented along uncorrelated coding directions (Supplementary Fig. 2), and the term *s* measures the amount of noise that lies along the signal directions. To see this, first note that the neural noise is described by the noise covariance matrix, cov(**x**∣**z**) = **Ψ** − **ΦΩ**^−1^**Φ**^T^, which appears in the definition of *s* above. Under the Gaussian model, this matrix measures the covariability of signal-unrelated, trial-to-trial fluctuations in pairs of units. On the other hand, the matrix cov(**x**) − cov(**x**∣**z**) = **ΦΩ**^−^^1^**Φ**^⊤^ measures the signal-related covariability of pairs of units, motivating its appearance in the definition of *f*^27,28^. The terms in the denominators of *s* and *f* give the projections of neural coding directions onto signal and noise subspaces, whereas the term ${\rm{Tr}}{(\mathbf{\Phi\Phi }^{\top })}^{2}$ acts as a normalization. Given that ‘neural noise’ likely includes variability that is related to variables that are not measured experimentally, we additionally describe, in the Supplementary Information, how these two factorization terms can be collapsed into a single factorization term.

### Gaussian simulations

To test our theory on data points that violate the assumptions of our theory, we began by sampling from a finite Gaussian model. Specifically, we drew latent vectors, **z**_*μ*_, from a multivariate Gaussian distribution whose covariance matrix had eigenvalues that decayed as a power law with rate *α*. Specifically, we set: *ω*_*i*_ = 5*i*^−*α*^, where *α* is the power law of the spectrum, and *ω*_*i*_ is the *i-*th eigenvalue of the latent covariance. The neural responses were then given by the formula **x**_*μ*_ = **Az**_*μ*_ for a random *n* × *d* Gaussian matrix **A** with i.i.d. elements or by applying a whitening transform, **x**_*μ*_ = **Ω**^−1/2^**z**_*μ*_. We set *n* = 80 and *d* = 40 for these simulations. For a fixed training set, we sampled *N*_task_ = 300 task **T** vectors and calculated the generalization error across all tasks using a set of new latent variables. Finally, we averaged over this entire procedure 30 times to generate the markers in Supplementary Fig. 1.

### Optimal codes

We derive which neuronal codes achieve the lowest generalization error, given a fixed number of samples to train on and a fixed latent structure. Specifically, we calculate which neuron–neuron and neuron–latent covariance matrices **Ψ** and **Φ** achieve the lowest multitask generalization error, given a fixed latent covariance **Ω** and training set size *p* (Supplementary Information). Note that, because we generate binary classification tasks by shattering the latent space uniformly, latent variables with more variance are, on average, more informative of the task labels than variables with low variance (Supplementary Information section 4). We perform this calculation by optimizing equation (5) with respect to **Ψ** and **Φ** subject to the constraint that the entire covariance matrix between neurons and latents be positive semi-definite, a necessary condition for the code to be realizable. Using this approach, we find that the left and right singular vectors of **Φ** are the eigenvectors of **Ψ** and **Ω**, respectively, for the optimal code. This shows that independent latent variables map directly onto uncorrelated directions in the firing rate space and that the principal components of the latent variables directly map onto the principal components of the neural activity (Supplementary Information). Furthermore, we obtain the following simple formula for the eigenvalues of the optimal neural code. Denoting the eigenvalues of **Ψ** and **Ω** as *ψ*_*i*_ and *ω*_*i*_, respectively, we have up to a permutation symmetry:

10 $${\psi }_{i}=C\frac{{\omega }_{i}}{2p{\omega }_{i}+\pi {\sum }_{k}{\omega }_{k}},$$

where *C* is an arbitrary constant. As *p* grows, we can see that the spectrum becomes flatter, reflecting the expansion strategy, whereas, as *p* shrinks, the spectrum decays faster and faster, reflecting the fact that less informative directions are compressed in the state space (Fig. 6c).

To validate our calculation, we numerically calculated the optimal code by optimizing on the space of positive semi-definite matrices. Because the full covariance matrix is positive semi-definite, there must exist matrices **X**_1_ and **X**_2_ such that

11 $$\mathbf{L}=\left(\begin{array}{rc}{\mathbf{\Omega} }^{1/2}&0\\ {\mathbf{X}}_{1}&{\mathbf{X}}_{2}\end{array}\right),\quad \mathbf{LL}^{T}=\left(\begin{array}{rc}\mathbf{\Omega} &{\mathbf{\Phi} }^{T}\\ \mathbf{\Phi} &\mathbf{\Psi} \end{array}\right)$$

The space of possible **X** matrices is unconstrained, so we simply optimize equation (5) with respect to the **X**_*i*_ matrices and calculate **Ψ** and **Φ** after the fact.

### MLP experiments

We used random and trained MLPs to test several predictions from our theory using explicitly non-Gaussian artificial neural response data. To generate these data, we first sampled a set of *d* = 40 dimensional latent variables from a multivariate Gaussian distribution with eigenvalues *ω*_*k*_ = *k*^−0.2^. For these experiments, we sampled 5 × 10^5^ latent variables independently from this distribution as a training dataset. Using these latent variables, we generated a set of *N*_*t*_ = 500 tasks by randomly shattering the latent space. Latent variables were then passed through a three-layer perceptron with randomly initialized weights. We chose the size of each intermediate layer to be twice the size of the previous one to ensure that the dimension of the representation did not decrease. Each layer was composed of a linear transform, followed by a ReLU nonlinearity (see Supplementary Figs. 5 and 6 for results using a tanh nonlinearity and Supplementary Fig. 7 for results using trained networks with an expanding layer structure). After sampling the task labels and passing the latents through the random MLP, we trained a three-hidden-layer MLP to predict task labels from the random MLP responses. The trained network was composed of linear-batchnorm-relu blocks, and we used the Adam optimizer to train the network^62^ through a single epoch. This setup corresponds to a multitask version of the hidden manifold modeling framework studied in deep learning theory^38^.

We evaluated the generalization error through layers and training using a new set of latents and tasks. Specifically, we sampled 10^3^ latent variables from the same distribution as well as a new set of 300 randomly selected tasks. To calculate the theoretical generalization error, we then used the network representations of these new latent variables to calculate the geometric metrics and evaluate the theoretical generalization errors using equation (5) with *p* = 300. To evaluate the empirical generalization error, we randomly split the new set of latents into a set of 300 training points and 700 test points. This process was repeated to calculate the average test error of the Hebbian rule across each layer and timepoint in training. Finally, we averaged over train/test splits to generate the markers shown in Fig. 3.

### Analysis of pose estimation network

To study the applicability of our theory to deep neural networks trained on complex data, we studied pose estimation networks trained using the DeepLabCut framework (version 2.3.10)^41,63^. We trained a pose estimation network based on a ResNet50 backbone^64^ using the default parameters of the DeepLabCut package for a total of 30,000 iterations. We trained this network on the ‘parenting mouse’ benchmark dataset^42^, which consists of 542 labeled frames (70% used for training the network and 30% for computing generalization error) containing an adult mouse with 12 body markers and two pups with five body markers each. We found that the test error was: 11.0 pixels, train: 3.1 pixels (image size varied but was a minimum of 704 × 480).

For simplicity, for all further analysis we examined only the 12 body markers of the adult mouse (*N* = 1) and only the 259 frames of the benchmark dataset in which all 12 of these markers were visible. Our *d* = 24 dimensional latent variables $\mathbf{z} \in {{\mathbb{R}}}^{24}$ consisted of the (*x*, *y*) coordinates (in pixels, normalized to an image size of 563 × 384) of the 12 body markers of the adult mouse. We also shifted the latents to have zero mean but did not rescale them. We extracted internal representations from the pose estimation network immediately before and immediately after each ReLU nonlinearity, resulting in a total of 33 different internal representations. For each of these 33 internal representations, we performed 20 random projections down to a fixed dimensionality of *n* = 100 to aid comparisons between layers of different sizes. All reported multitask errors and geometric measures were averaged over these 20 random projections. The internal representations $\mathbf{x}\in {{\mathbb{R}}}^{100}$ were also shifted to have zero mean but were not rescaled.

### Macaque analyses

We drew from a publicly available dataset containing multi-unit recordings from V4 and IT taken from two monkeys^43^ via the Brain-Score package^65,66^. These recordings were taken as the monkeys viewed visual stimuli as described in ref. ^43^ and contained 88 V4 and 168 IT neural sites, although we reproduce all results projecting IT and pixel responses down to 88 randomly chosen dimensions (Supplementary Fig. 8). The stimuli for this task were drawn from a generative model in which a total of 64 objects coming from eight categories were displayed against varying backgrounds. These images were generated by varying *d* = 6 continuous latent variables that controlled the object size, angle and position. Latent variables were drawn randomly from a uniform distribution.

We tested the linear decodability of these latent variables from the neural firing rates using the scheme described in Methods ‘Model of multi-task learning’. For the raw pixels, we first carried out a Gaussian random projection onto a 500-dimensional space before applying this scheme. Specifically, for each of the eight object category types, we formed task labels for *N*_*t*_ = 300 tasks by randomly shattering the latent space. There were 320 examples per category type. We *z*-scored the latent variables to ensure that there was no especially informative variable. We then formed predictions using the supervised Hebbian readout described in Methods ‘Model of multi-task learning’ after mean centering the neuronal firing rates. This procedure was repeated over 15 different train/test data splits. To generate the results in Fig. 5, we then averaged the generalization error across the *N*_*t*_ classification tasks, data splits and image categories. To test the significance of the differences across groups, we used paired-sample *t*-tests across each of the eight category types and adjusted using a false discovery rate (FDR) criterion using the statsmodels package (Supplementary Table 1). In Supplementary Information, we also show the geometry and generalization errors for each of the categories individually (Supplementary Figs. 9 and 10) as well as the error obtained by pooling all categories together (Supplementary Fig. 11).

### Spatial representations in rat PFC and CA1

#### Rat data processing

We drew from a dataset reported in ref. ^45^, which is publicly available^67^. In brief, rats learned to perform a continuous alternation task in a W-maze that required them to travel to the center of the maze and then to the opposite end from the one they began from (Fig. 7b). Learning occurred over eight sessions lasting approximately 20 minutes each. The position of each animal was measured at 30 fps using an LED light attached to the animal’s head. Neural recordings were collected from multi-tetrodes implanted in dorsal CA1 and medial PFC, and we used the preprocessed spike-sorted data provided in ref. ^67^.

For the analyses reported here, we ran several additional preprocessing steps. First, the activity of putative single units was binned at 500 ms. To be included in the analyses for a given session, a unit had to achieve a mean firing rate of 0.1 Hz during that session. To align the behavioral position data to spike bins, we used Nadaraya–Watson kernel smoothing with a Gaussian kernel to interpolate the reported position information. We similarly calculated *x* and *y* velocities using the displacements in the animal’s position, followed by Nadaraya–Watson interpolation. For all analyses here, we only considered timepoints when the animal had an overall speed of at least 4 cm s^−1^ (ref. ^45^).

#### Generalized linear model analysis

In Fig. 7g, we showed that the total neural dimensionality decreases over the initial four sessions. This initial decrease in dimension can come from reductions in navigation-unrelated directions, signal directions or a mix of the two. In the main text, we argued that this initial drop in the total dimension comes from a compression of navigation-unrelated directions. To support this interpretation, we showed that the dimension of the subspace containing task-relevant information (that is, the dimension of the ‘signal subspace’) increases monotonically over learning, even during those initial four sessions (Fig. 7h). This suggests that any drop in the total dimensionality comes from a compression of navigation-unrelated directions. The monotonic increase of the task-relevant dimensionality through learning is in line with our predictions regarding optimal representations. We begin by giving a brief summary of this analysis, before describing the details.

To estimate the dimension of the subspace containing task-relevant information, we require a method to average out those components of neuronal activity that are unrelated to the task—in this case, unrelated to the navigational state of the animal. Formally, one requires

12 $$\mathbf{v}(\mathbf{z}):= {{\mathbb{E}}}_{\mathbf{x}}[\mathbf{x}|\mathbf{z}],$$

where **z** is the vector of latents describing the navigational state of the animal and *x* are the firing rates. To estimate this conditional expectation, we used Poisson generalized linear models (GLMs). The dimension of the task-related subspace follows from the participation ratio of **v**(**z**):

13 $${\rm{dim.}}\,{\rm{task}}\,{\rm{subspace}}={\rm{PR}}({\rm{cov}}(\mathbf{v})).$$

This is the quantity we plot in Fig. 7h, after *z*-scoring **v**.

We used Poisson GLMs to predict single-unit spike counts from position and velocity behavioral variables. To do this, we used isotropic Gaussian basis functions to tile the two-dimensional maze coordinates and one-dimensional Gaussian basis functions to tile the *x* and *y* velocity information of the animal. We then fit coefficients that predicted spike count probabilities using a linear combination of the values of these basis functions that was then fed through an exponential link function. To accommodate idiosyncrasies in each animal’s movement preferences, we adaptively chose the locations of each basis function separately for each animal. Basis function centers were determined by binning the distribution of *x* and *y* coordinates for each animal across all sessions and using the center of bins that contained data from more than 20 time bins. To accommodate for the possibility of trajectory-specific place maps, we fit separate coefficients for these basis functions for each trajectory type (inbound versus outbound). We formed basis functions for the velocity variables by separately tiling each one-dimensional space of *x* and *y* velocities using a similar adaptive binning procedure. All GLM analyses were carried out using the PoissonRegressor scikitlearn class.

All GLM model hyperparameters were set by cross-validation. Our models had an *ℓ*2 regularization hyperparameter, together with four hyperparameters governing the distribution of basis functions: the bin sizes for the velocity variables, the bin sizes for the position variables and the variances of the corresponding basis functions. We set *ℓ*2 regularization parameters separately for each unit using 10-fold cross-validation over 10 possible regularization parameter values. We chose a single set of basis function hyperparameters across rats. To choose the basis hyperparameters, we searched over a small grid and chose the combination that achieved the smallest cross-validated generalization error. The cross-validated error for each single-unit model was calculated using the default percentage of deviance explained. The average cross-validated population *D*^2^ across all sessions and rats was 0.29 in PFC and 0.25 in CA1, and the standard deviation of the session-averaged population *D*^2^ across rats was 0.022 in PFC and 0.1 in CA1. Single-unit models that performed worse than a constant firing rate null model (*D*^2^ < 0) were discarded and replaced by the null model—that is, were treated as having an average firing rate that did not depend on the latent variables.

We used these GLMs to estimate the dimensionality of the component of neural activity related to navigational signals. Just as with previous analyses, we calculated this dimensionality using the participation ratio of the covariance matrix—in this case, the covariance of expected neuronal firing rates, $\mathbf{v}(\mathbf{z})= {\mathbb{E}}_{\mathbf{x}}[\mathbf{x}|\mathbf{z}].$ The covariance matrix was given by ${{\mathbb{E}}}_{\mathbf{z}}\left[\mathbf{v}(\mathbf{z})\mathbf{v}{(\mathbf{z})}^{\top }\right]-\left({{\mathbb{E}}}_{\mathbf{z}}\mathbf{v}(\mathbf{z})\right){\left({{\mathbb{E}}}_{\mathbf{z}}\mathbf{v}(\mathbf{z})\right)}^{\top }$, and the expectation over **z** was calculated using the empirical distribution of latent variables within a given session. (Note that we *z*-scored the **v** vector to match our treatment of the raw neural data as described below).

#### Readout analysis

Before carrying out the linear readout analysis of the spiking data, we normalized both behavioral variables and spike count data. We centered each unit using its mean from a session and divided by the unit’s standard deviation within a session plus a small regularizer of 0.02 Hz. This regularizer was added to avoid massively overweighting very sparse units, and modifying its precise value did not significantly affect our results. Within the multitask learning framework considered here, the importance of a given latent variable depends on its variance (‘Optimal representation of latent variables’ and Supplementary Information section 4). Hence, we *z*-scored the behavioral variables so that all position and velocity variables would be weighted equally when forming random binary classification tasks. Finally, because each rat and session had different numbers of trials and usable units, we calculated the geometric terms from random subsets of neurons and trials. To use all available rat data while balancing across rats and sessions, we used 19 PFC units and 24 CA1 units. For each random neuronal subset, we chose a random subset of 500 trials (*p* = 300 training points) to use for the readout and geometric analysis. All quantities reported in the main text and the Supplementary Information correspond to an average over 500 such subsets.

To test the significance of our trends, we fit quadratic regression models with mixed-effect intercepts to each geometric measure over time. That is, a given geometric quantity *y* during session *t* for rat *i* was modeled as *y*_*it*_ = *β*_*i*_ + *γt* + *ηt*^2^. In the Supplementary Information, we plot model fits and report both the parameter values and *P* values of these quantities, which we calculated using the statsmodels Python package (Supplementary Fig. 12 and Supplementary Table 2).

### Statistics and reproducibility

We provide publicly available code to reproduce our results. We did not collect any data for this study, so no statistics were used to determine sample size. We excluded neurons with extremely low firing rates from the analyses presented in the ‘Geometry of spatial representations in PFC and CA1 during learning’. We additionally excluded frames in which body markers were invisible in ‘Disentangling in deep pose estimation networks’ Finally, for the analyses in ‘Predicting readout performance of macaque visual representations’, we excluded noisy units, following the recommendations in the Brain-Score package^65,66^.

### Reporting summary

Further information on research design is available in the Nature Portfolio Reporting Summary linked to this article.

## Online content

Any methods, additional references, Nature Portfolio reporting summaries, source data, extended data, supplementary information, acknowledgements, peer review information; details of author contributions and competing interests; and statements of data and code availability are available at 10.1038/s41593-025-02183-y.

## Supplementary information

Supplementary Information (1) Proof of generalization error formula. (2) Gaussian simulations (Supplementary Fig. 1). (3) Decomposition of generalization error into geometric terms (Supplementary Fig. 2). (4) Optimal geometry calculation. (5) SVC comparison (Supplementary Fig. 3). (6) Additional MLP analyses (Supplementary Figs. 4–7). (7) Additional macaque analyses (Supplementary Figs. 8–11 and Supplementary Table 1). (8) Additional rat analyses (Supplementary Fig. 12 and Supplementary Table 2).

Reporting Summary

## Data Availability

All macaque data are publicly available from Brain-Score^65,66^ at https://github.com/brain-score/vision. Rat data are available from the DANDI Archive^67^. Mouse pose data are available from DeepLabCut^41,63^ at github.com/DeepLabCut/DeepLabCut.

## Code availability

All code and results used in this paper are publicly available at https://github.com/awakhloo/population_geometry_optimal_coding.
