# Supplementary material for: Neural population geometry and optimal coding of tasks with shared latent structure
Source: Nat Neurosci. 2026 Feb 4;29(3):682–92. doi: 10.1038/s41593-025-02183-y (PMC12971491; doi:10.1038/s41593-025-02183-y)
Supplement: Supplementary file 1 — (1) Proof of generalization error formula. (2) Gaussian simulations (Supplementary Fig. 1). (3) Decomposition of generalization error into geometric terms (Supplementary Fig. 2). (4) Optimal geometry calculation. (5) SVC comparison (Supplementary Fig. 3). (6) Additional MLP analyses (Supplementary Figs. 4–7). (7) Additional macaque analyses (Supplementary Figs. 8–11 and Supplementary Table 1). (8) Additional rat analyses (Supplementary Fig. 12 and Supplementary Table 2). [file 41593_2025_2183_MOESM1_ESM.pdf]

nature neuroscience

Supplementary information

https://doi.org/10.1038/s41593-025-02183-y

Neural population geometry and optimal coding of tasks with shared latent structure

In the format provided by the authors and unedited

Contents

1 Proof of generalization error formula 1

2 Validation on Gaussian simulations 8

3 Decomposition of generalization error into geometric terms 9

4 Optimal geometry 12

5 Comparison of all analyses to SVC 17

6 Additional MLP analyses 18

7 Additional macaque analyses 21

8 Additional rat analyses 24

1 Proof of generalization error formula

Here we state and prove our formula for the generalization error.

Theorem 1. *Let $x_{n,\mu} \in \mathbb{R}^n, z_{n,\mu} \in \mathbb{R}^d$ with $1 \leq \mu \leq p$ be a sequence of random variables drawn independently from a Gaussian distribution with mean zero and covariance matrices:*

$$C_n = \begin{pmatrix} \Psi_n & \Phi_n \\ \Phi_n^\top & \Omega_n \end{pmatrix}, \tag{1}$$

where C_n is a positive-definite matrix with sub-matrices $\Psi_n \in \mathbb{R}^{n\times n}, \Omega_n \in \mathbb{R}^{d\times d}, \Phi_n \in \mathbb{R}^{n\times d}$, and the ratios $d/n = \alpha$ and $p/n = \beta$ are held fixed. In addition, let $x_{n,+}, z_{n,+}$ be a pair of samples drawn independently from the same distribution, and let r_n be a sequence of d-dimensional teacher vectors drawn uniformly from the surface of the sphere of radius $\sqrt{d}$. Assume that there exist positive constants c_1, c_2 such that

$$\frac{c_1}{n} \leq \psi_{n,i}, \omega_{n,i} \leq \frac{c_2}{n}, \tag{2}$$

$$0 \leq \phi_{n,i} \leq \frac{c_2}{n}, \tag{3}$$

where we use lower-case Greek letters to denote the eigenvalues of Ψ_n and Ω_n and the singular values of Φ_n. Define the Hebbian readout $w := \frac{1}{p}\sum_\mu \operatorname{sgn}(\langle z_{n,\mu}, r_n\rangle)x_{n,\mu}$, and let $\Theta(\cdot)$ denote the Heaviside step function. Under these assumptions the generalization error is given by

$$\begin{aligned} &\mathbb{E}_{x_n,z_n,r_n}\left\{\Theta(-\operatorname{sgn}(\langle r_n, z_{n,+}\rangle)\langle w, x_{n,+}\rangle)\right\} \qquad (4)\\ &= \frac{1}{\pi}\tan^{-1}\left(\sqrt{\frac{\operatorname{Tr}(\Omega_n)\left[\frac{\pi}{p}\operatorname{Tr}(\Psi_n^2)\operatorname{Tr}(\Omega_n) + 2\operatorname{Tr}(\Phi_n^\top\Psi_n\Phi_n)\right]}{2\operatorname{Tr}(\Phi_n^\top\Phi_n)^2} - 1}\right) + O(n^{-1/2}). \end{aligned}$$

Proof. We start by defining the random variables

$$\gamma_n^\mu = \mathrm{sgn}(\langle z_{n,\mu}, r_n\rangle)\langle x_{n,+}, x_{n,\mu}\rangle, \tag{5}$$

so that the generalization error may be written

$$\mathbb{E}_{r_n}\mathbb{E}_{x_{n,+},z_{n,+}}\mathbb{E}_{x_{n,\mu},z_{n,\mu}}\Theta\biggl(- \mathrm{sgn}(\langle z_{n,+}, r_n\rangle)\sum_\mu \gamma_n^\mu\biggr). \tag{6}$$

Note that we have used Fubini's theorem to separate the expectations over the training and test points, as well as the teacher vector r_n.

Our goal is now to show that the distribution of the random variable $\sum_\mu \gamma_n^\mu$ is approximately Gaussian. This allows us to carry out the inner expectation in Eq. 6. We do this using the Berry-Esseen theorem. We can obtain the relevant moments needed to apply this theorem using the identity

$$\mathbb{E}_{p,q\sim\mathcal{N}(0,\Sigma)}\mathrm{sgn}(q)p = \sqrt{\frac{2}{\pi\sigma}}\kappa, \quad \Sigma := \begin{pmatrix}\zeta & \kappa\\ \kappa & \sigma\end{pmatrix}, \tag{7}$$

together with

$$\mathbb{E}_{h\sim\mathcal{N}(0,v)}|h|^3 = \frac{2\sqrt{2}}{\sqrt{\pi}}v^{3/2}. \tag{8}$$

The mean and variance are simply

$$\mathbb{E}_{x_{n,\mu},z_{n,\mu}}\gamma_n^\mu = \sqrt{\frac{2}{\pi r_n^\top \Omega_n r_n}}x_{n,+}^\top\Phi_n r_n, \tag{9}$$

$$\mathbb{E}_{x_{n,\mu},z_{n,\mu}}(\gamma_n^\mu - \mathbb{E}_{x_{n,\mu},z_{n,\mu}}\gamma_n^\mu)^2 = x_{n,+}^\top\Psi_n x_{n,+} - \frac{2(x_{n,+}^\top\Phi_n r_n)^2}{\pi r_n^\top\Omega_n r_n}. \tag{10}$$

It suffices to bound the third central moment as

$$\mathbb{E}_{x_{n,\mu},z_{n,\mu}}|\gamma_n^\mu - \mathbb{E}_{x_{n,\mu},z_{n,\mu}}\gamma_n^\mu|^3 \le 4\biggl(\frac{2\sqrt{2}}{\sqrt{\pi}}\bigl(x_{n,+}^\top\Psi_n x_{n,+}\bigr)^{3/2} + |\mathbb{E}_{x_{n,\mu},z_{n,\mu}}\gamma_n^\mu|^3\biggr). \tag{11}$$

We now define the following random variables:

$$\sigma_n := r_n^\top\Omega_n r_n, \quad \kappa_n := x_{n,+}^\top\Phi_n r_n, \quad \zeta_n := x_{n,+}^\top\Psi_n x_{n,+}, \quad \eta_n := z_{n,+}^\top r_n. \tag{12}$$

If we now let $F_n(s)$ denote the cumulative distribution function of the standardized sum

$$s = \frac{\sum_\mu (\gamma_n^\mu - \mathbb{E}_{x_{n,\mu}} \gamma_n^\mu)}{\sqrt{p(\zeta_n - \frac{2\kappa_n^2}{\pi\sigma_n})}} \tag{13}$$

and denote by $N(s)$ the standard normal c.d.f., the Berry-Esseen theorem [3] gives

$$\Big| F_n(s) - N(s) \Big| \leq \frac{K}{\sqrt{p}\big(1 - \frac{2\kappa_n^2}{\pi\sigma_n\zeta_n}\big)^{3/2}} \big(1 + (x_{n,+}^\top \Psi_n x_{n,+})^{-3/2} |\mathbb{E}_{x_{n,\mu}, z_{n,\mu}} \gamma_n^\mu|^3\big), \tag{14}$$

where K is a constant. We can see that the rightmost term contributes at most a constant factor from

$$\frac{|\mathbb{E}_{x_{n,\mu}, z_{n,\mu}} \gamma_n^\mu|^3}{(x_{n,+}^\top \Psi_n x_{n,+})^{3/2}} \leq \frac{M |x_{n,+}^\top \Phi_n r_n|^3}{(r_n^\top \Omega_n r_n)^{3/2} (x_{n,+}^\top \Psi_n x_{n,+})^{3/2}} \leq M' \frac{\phi_{n,\max}^3}{\omega_{n,\min}^{3/2} \psi_{n,\min}^{3/2}} = O(1), \tag{15}$$

for some positive constants M, M'. Next we show that the entire right hand side of Eq. (14) is $O(n^{-1/2})$ using the following Lemma.

Lemma 2. *Let C be a positive definite matrix with submatrices*

$$C = \begin{pmatrix} \Psi & \Phi \\ \Phi^\top & \Omega \end{pmatrix}, \tag{16}$$

with $\Psi \in \mathbb{R}^{n\times n}, \Omega \in \mathbb{R}^{d\times d}$, and $\Phi \in \mathbb{R}^{n\times d}$. Then for all $h \in \mathbb{R}^n$ and $r \in \mathbb{R}^d$

$$\frac{(h^\top \Phi r)^2}{h^\top \Psi h r^\top \Omega r} \leq 1. \tag{17}$$

Proof. Let (x, z) be mean-zero jointly Gaussian vectors with covariance matrix C. It follows that

$$(h^\top \Phi r)^2 = \big(\mathbb{E}[\langle x, h\rangle \langle r, z\rangle]\big)^2 \tag{18}$$
$$\leq \mathbb{E}[\langle x, h\rangle^2] \mathbb{E}[\langle z, r\rangle^2] \tag{19}$$
$$= (h^\top \Psi h)(r^\top \Omega r). \tag{20}$$

□

We therefore obtain

$$\frac{2\kappa_n^2}{\pi\sigma_n\zeta_n} \leq \frac{2}{\pi}, \tag{21}$$

so that the right hand side of Eq. (14) is $O(n^{-1/2})$. Keeping only the leading order terms, we obtain the following expression for the generalization error

$$\frac{1}{2}\mathbb{E}_{r_n}\mathbb{E}_{x_n,z_n}\mathrm{erfc}\left(\frac{\mathrm{sgn}(\eta_n)\kappa_n}{\sqrt{\frac{\pi}{p}\sigma_n\zeta_n(1-\epsilon)}}\right)+O(n^{-1/2}), \tag{22}$$

$$\epsilon := \frac{2\kappa_n^2}{\pi\sigma_n\zeta_n}. \tag{23}$$

Note that we have dropped the + subscript from the test point and latent pair. We can deal with the term $\epsilon \leq 2/\pi$ by noting that

$$\mathbb{E}_{x_n,r_n}\frac{2\kappa_n^2}{\pi\sigma_n\zeta_n} \leq \mathbb{E}_{s,r_n}\frac{2(s^\top\Psi^{1/2}\Phi_n r_n)^2}{\pi d\psi_{n,\min}\omega_{n,\min}} = O(n^{-1}), \tag{24}$$

where $\psi_{n,\min}, \omega_{n,\min}$ are the smallest eigenvalues of Ψ_n, Ω_n, and s is a random vector drawn uniformly from the surface of the unit sphere. From here we can simply expand to first order to see that the ϵ term contributes at most $O(n^{-1})$.

We now show that we may replace the quadratic form involving x_n with its expected value. To do this, we first note that we can focus on estimating the expectation over a region which in which $|\zeta_n - \mathrm{Tr}(\Psi_n^2)| < c\mathrm{Tr}(\Psi_n^2)$ for some $0 < c < 1$ by applying the Hanson-Wright inequality. This gives

$$\left|\mathbb{E}_{r_n,x_n,z_n}\left[\mathrm{erfc}\left(\frac{\mathrm{sgn}(\eta_n)\kappa_n}{\sqrt{\frac{\pi}{p}\sigma_n\mathrm{Tr}(\Psi_n^2)}}\right)-\mathrm{erfc}\left(\frac{\mathrm{sgn}(\eta_n)\kappa_n}{\sqrt{\frac{\pi}{p}\sigma_n\zeta_n}}\right)\right]\right| \tag{25}$$

$$\leq \mathbb{E}_{r_n,x_n,z_n}\mathbf{1}_{\{|\zeta_n-\mathrm{Tr}(\Psi_n^2)|<c\mathrm{Tr}(\Psi_n^2)\}}\left|\mathrm{erfc}\left(\frac{\mathrm{sgn}(\eta_n)\kappa_n}{\sqrt{\frac{\pi}{p}\sigma_n\mathrm{Tr}(\Psi_n^2)}}\right)-\mathrm{erfc}\left(\frac{\mathrm{sgn}(\eta_n)\kappa_n}{\sqrt{\frac{\pi}{p}\sigma_n\zeta_n}}\right)\right|+O(e^{-g\sqrt{n}}),$$

for some constant g that depends on the choice of c. We can then obtain the following bound for the integrand in the region where $|\zeta_n - \mathrm{Tr}(\Psi_n^2)| < c\mathrm{Tr}(\Psi_n^2)$ by the mean value theorem:

$$\mathbb{E}_{r_n,x_n,z_n}\mathbf{1}_{\{|\zeta_n-\mathrm{Tr}(\Psi_n^2)|<c\mathrm{Tr}(\Psi_n^2)\}}\left|\mathrm{erfc}\left(\frac{\mathrm{sgn}(\eta_n)\kappa_n}{\sqrt{\frac{\pi}{p}\sigma_n\mathrm{Tr}(\Psi_n^2)}}\right)-\mathrm{erfc}\left(\frac{\mathrm{sgn}(\eta_n)\kappa_n}{\sqrt{\frac{\pi}{p}\sigma_n\zeta_n}}\right)\right| \tag{26}$$

$$\leq \mathbb{E}\frac{\sqrt{p}C|\kappa_n|}{\mathrm{Tr}(\Psi_n^2)^{3/2}}\left|\zeta_n-\mathrm{Tr}(\Psi_n^2)\right| \leq \frac{\sqrt{p}C\sqrt{\mathbb{E}[\kappa_n^2]\mathbb{E}|\zeta_n-\mathrm{Tr}(\Psi_n^2)|^2}}{\mathrm{Tr}(\Psi_n^2)^{3/2}}. \tag{27}$$

for some constant C. We can now use the following Lemma to show that the right hand side is $O(n^{-1/2})$ in expectation:

Lemma 3. *Let $r \in \mathbb{R}^d$ be a vector distributed either uniformly on the surface of the sphere of radius $\sqrt{d}$ or according to a mean-zero Gaussian with covariance I_d. Then for any matrix $A \in \mathbb{R}^{d\times d}$ we have*

$$\mathbb{E}|r^\top Ar - \mathrm{Tr}(A)| = O(||A||_{\mathrm{op}}) + O(||A||_F), \tag{28}$$

$$\mathbb{E}|r^\top Ar - \mathrm{Tr}(A)|^2 = O(||A||_{\mathrm{op}}^2) + O(||A||_F^2) \tag{29}$$

where $||\cdot||_{\mathrm{op}}$ is the operator norm induced by the Euclidean norm.

Proof. Applying the Hanson-Wright inequality for random vectors satisfying the convex concentration property [1, 5] we obtain the first identity

$$\int p(dr)|r^\top Ar - \mathrm{Tr}(A)| = \int_0^\infty dt\mathbb{P}(|r^\top Ar - \mathrm{Tr}(A)| > t) \tag{30}$$

$$\leq \int_0^\infty dt \exp\bigg(-\frac{Ct^2}{||A||_F^2}\bigg) + \exp\bigg(-\frac{ct}{||A||_{\mathrm{op}}}\bigg) = O(||A||_F) + O(||A||_{\mathrm{op}}), \tag{31}$$

where C, c are positive constants. Similarly we have

$$\int p(dr)|r^\top Ar - \mathrm{Tr}(A)|^2 = 2\int_0^\infty dt\mathbb{P}(|r^\top Ar - \mathrm{Tr}(A)| > t)t = O(||A||_F^2) + O(||A||_{\mathrm{op}}^2), \tag{32}$$

yielding the second identity. □

Applying this estimate and using the fact that $\mathbb{E}\kappa_n^2 = \mathrm{Tr}(\Phi_n^\top \Psi_n \Phi_n) = O(n^{-2})$ then shows that the right hand side is $O(n^{-1/2})$. We are therefore left with

$$\frac{1}{2}\mathbb{E}_{r_n}\mathbb{E}_{\eta_n,\kappa_n}\mathrm{erfc}\Bigg(\frac{\mathrm{sgn}(\eta_n)\kappa_n}{\sqrt{\frac{\pi}{p}\sigma_n \mathrm{Tr}(\Psi_n^2)}}\Bigg) + O(n^{-1/2}). \tag{33}$$

From our definitions, the variables η_n, κ_n follow a bivariate Gaussian distribution:

$$\eta_n, \kappa_n \sim \mathcal{N}\Bigg(0, \begin{pmatrix} r_n^\top \Omega_n r & r_n^\top \Phi_n^\top \Phi_n r_n \\ r_n^\top \Phi_n^\top \Phi_n r_n & r_n^\top \Phi_n^\top \Psi_n \Phi_n r_n \end{pmatrix}\Bigg). \tag{34}$$

The inner expectation can now be carried out analytically. We start by integrating over the conditional distribution $\kappa_n|\eta_n$, which is Gaussian with mean $\eta_n r_n^\top \Phi_n^\top \Phi_n r/\sigma_n$ and variance $r_n^\top \Phi_n^\top \Psi_n \Phi_n r_n - (r_n^\top \Phi_n^\top \Phi_n r_n)^2/\sigma_n$. To do this, we need the Gaussian integral over the complementary error function

$$\frac{1}{2}\int \frac{dx}{\sqrt{2\pi v}} e^{-(x-m)^2/(2v)}\mathrm{erfc}(cx) = \frac{1}{2}\mathrm{erfc}\bigg(\frac{mc}{\sqrt{1+2vc^2}}\bigg). \tag{35}$$

This formula can be derived by considering the function

$$I(a, b) := \int Dx \mathrm{erfc}(ax + b), \tag{36}$$

where Dx is a standard Gaussian measure. This integral can be solved by differentiating under the integral with respect to b, leading to Eq. (35). Applying this identity to Eq. (33) we obtain

$$\frac{1}{2}\mathbb{E}_{r_n}\mathbb{E}_{\eta_n}\mathrm{erfc}\Bigg(\frac{|\eta_n| r_n^\top \Phi_n^\top \Phi_n r_n}{r_n^\top \Omega_n r_n \sqrt{\frac{\pi}{p}\sigma_n \mathrm{Tr}(\Psi_n^2) + 2\big[r_n^\top \Phi_n^\top \Psi_n \Phi_n r_n - \frac{(r_n^\top \Phi_n^\top \Phi_n r_n)^2}{r_n^\top \Omega_n r_n}\big]}}\Bigg). \tag{37}$$

We can now split the integral over the η_n into half Gaussian integrals over complementary error functions. This allows us to invoke the relation

$$\frac{1}{2}\int_0^\infty \frac{\sqrt{2}dx}{\sqrt{\pi}} e^{-x^2/2}\mathrm{erfc}(cx) = \frac{1}{\pi}\tan^{-1}\left(\frac{1}{\sqrt{2}c}\right). \tag{38}$$

This formula can similarly be derived by considering the function

$$J(c) := \int_0^\infty Dx\mathrm{erfc}(cx) \tag{39}$$

and differentiating with respect to c. This leaves

$$\frac{1}{\pi}\mathbb{E}_{r_n}\tan^{-1}\left(\sqrt{\frac{r_n^\top\Omega_n r_n\left[\frac{\pi}{p}\mathrm{Tr}(\Psi_n^2)r_n^\top\Omega_n r_n + 2r_n^\top\Phi_n^\top\Psi_n\Phi_n r_n\right]}{2(r_n^\top\Phi_n^\top\Phi_n r_n)^2}-1}\right) + O(n^{-1/2}). \tag{40}$$

Note that *this gives the generalization error for a fixed task vector r_n.*

To carry out the final expectation, we expand these quadratic forms around their mean values. To do this, let us introduce the $O(1)$ variables

$$\sigma_1^n = r_n^\top\Omega_n r_n, \quad \sigma_2^n = n^2 r_n^\top\Phi_n^\top\Psi_n\Phi_n r_n, \quad \sigma_3^n = n r_n^\top\Phi_n^\top\Phi_n r_n, \tag{41}$$

and the function

$$G(\sigma^n) = \tan^{-1}\left(\sqrt{\frac{\sigma_1^n(c\sigma_1^n + 2n^{-2}\sigma_2^n)}{2n^{-2}(\sigma_3^n)^2}-1}\right), \tag{42}$$

where $c := \frac{\pi}{p}\mathrm{Tr}(\Psi_n^2) = O(n^{-2})$. We start by noting that we can focus on estimating the expectation over a region in which $||\sigma^n - \mathbb{E}\sigma^n|| < t$ for $t > 0$. To see this, note that by the Hanson-Wright inequality:

$$\mathbb{E}_{r_n}G(\sigma^n) = \mathbb{E}_{r_n}\mathbf{1}_{\{||\sigma^n-\mathbb{E}\sigma^n||<t\}}G(\sigma^n) + O(e^{-c\sqrt{n}}). \tag{43}$$

We can now bound the remaining error incurred by replacing σ^n with its expected value as:

$$|G(\sigma^n) - G(\mathbb{E}\sigma^n)| \leq \sum_i \sup_{||\xi-\mathbb{E}\sigma^n||<t} |\partial_i G(\xi)||\sigma_i^n - \mathbb{E}\sigma_i^n| \tag{44}$$

$$= \sum_i \sup_{||\xi-\mathbb{E}\sigma^n||<t}\left|\left[\frac{\xi_1(c\xi_1+2n^{-2}\xi_2)}{2n^{-2}(\xi_3)^2}-1\right]^{-1/2}\left[\frac{\xi_1(c\xi_1+2n^{-2}\xi_2)}{2n^{-2}(\xi_3)^2}\right]^{-1}\right. \tag{45}$$

$$\tag{46}$$

$$\left.\partial_i\frac{\xi_1(c\xi_1+2n^{-2}\xi_2)}{2n^{-2}(\xi_3)^2}\right||\sigma_i^n - \mathbb{E}\sigma_i^n| \tag{47}$$

$$\leq M \sup_{||\xi-\mathbb{E}\sigma^n||<t}\left|\frac{\xi_1(c\xi_1+2n^{-2}\xi_2)}{2n^{-2}(\xi_3)^2}-1\right|^{-1/2}\sum_i|\sigma_i^n - \mathbb{E}\sigma_i^n|, \tag{48}$$

for some constant M. We can now use the following lemma to give a uniform bound on the remaining term:

Lemma 4. *Let C be a positive definite matrix with submatrices*

$$C = \begin{pmatrix} \Psi & \Phi \\ \Phi^\top & \Omega \end{pmatrix}, \tag{49}$$

with $\Psi \in \mathbb{R}^{n\times n}, \Omega \in \mathbb{R}^{d\times d}$, and $\Phi \in \mathbb{R}^{n\times d}$. Then

$$\frac{\mathrm{Tr}(\Omega)\mathrm{Tr}(\Phi^\top \Psi \Phi)}{\mathrm{Tr}(\Phi^\top \Phi)^2} \geq 1. \tag{50}$$

Proof. The claim follows from an application of the Cauchy-Schwarz inequality:

$$\mathrm{Tr}(\Phi^\top \Phi)^2 = (\mathbb{E}\langle \Phi^\top x, z\rangle)^2 \leq \mathbb{E}||\Phi^\top x||^2 \mathbb{E}||z||^2 = \mathrm{Tr}(\Phi^\top \Psi \Phi)\mathrm{Tr}(\Omega), \tag{51}$$

where the expectation is taken over zero-mean, jointly Gaussian vectors (x, z) which have a covariance matrix C. □

From this lemma and our assumptions on the spectrum, it follows that there exists a constant $M' > 0$ that does not depend on n such that

$$\frac{\langle \sigma_1^n \rangle (c\langle \sigma_1^n \rangle + 2n^{-2}\langle \sigma_2^n \rangle)}{2n^{-2}\langle \sigma_3^n \rangle^2} \geq 1 + M', \tag{52}$$

where we use brackets to denote the expectation. Hence if we choose t sufficiently small, we obtain

$$|G(\sigma^n) - G(\mathbb{E}\sigma^n)| \leq M'' \sum_i |\sigma_i^n - \mathbb{E}\sigma_i^n|, \tag{53}$$

where M'' is a positive constant. By Lemma 3, the right hand side is $O(n^{-1/2})$.

Replacing these quadratic forms with their means, we obtain the final result:

$$\frac{1}{\pi} \tan^{-1} \left(\sqrt{\frac{\mathrm{Tr}(\Omega_n) \left[\frac{\pi}{p} \mathrm{Tr}(\Psi_n^2) \mathrm{Tr}(\Omega_n) + 2\mathrm{Tr}(\Phi_n^\top \Psi_n \Phi_n) \right]}{2\mathrm{Tr}(\Phi_n^\top \Phi_n)^2} - 1} \right) + O(n^{-1/2}). \tag{54}$$

□

Remark. *Note that if one was to consider a non-isotropic Gaussian distribution over teacher vectors r, this would lead to a non-uniform shattering of the latent space. Supposing r follows a multivariate Gaussian distribution with covariance matrix Σ, one can see from Eq. (6) that this setting is equivalent to changing the covariance of z via the transformation $\Omega \mapsto \Sigma^{1/2}\Omega\Sigma^{1/2}$. To give a concrete example, if one takes the covariance matrix of r to be diagonal with elements $(v_1, \ldots, v_d)$, then this is equivalent to rescaling each latent dimension by a factor of $\sqrt{v_i}$. Thus, this would be equivalent to considering a distribution of binary classification problems that emphasize those dimensions with larger v_i.*

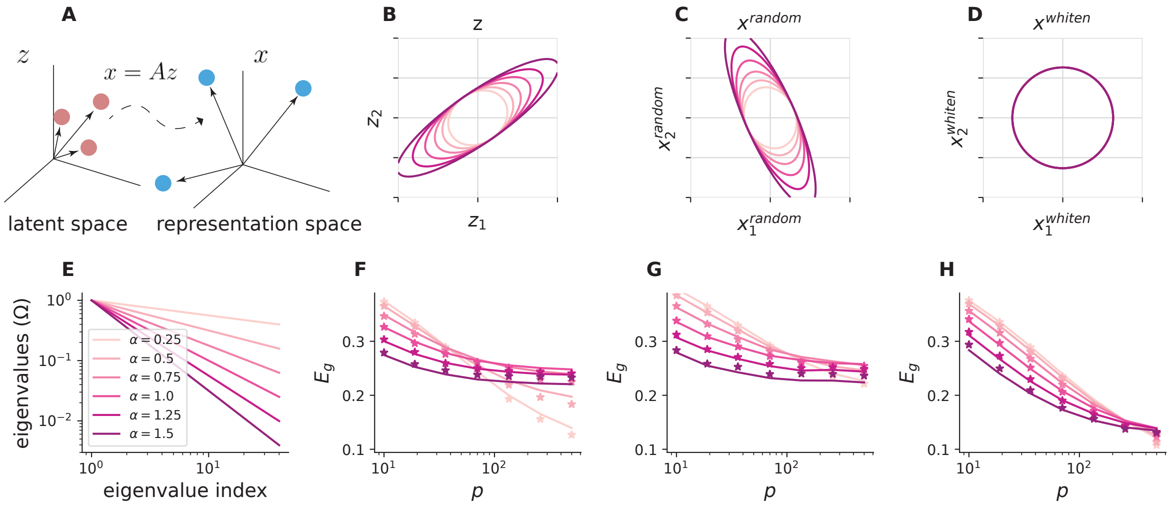

Figure 1: Theory predicts empirical generalization error in Gaussian model with power law covariance spectra. (a) Schematic illustrating simulation setup. Gaussian latent variables z are used to generate task labels, and predictions are formed using a linear transformation of the latent variables, $x = Az$. (b-d) Two typical units for the (b) latent variables, (c) random high dimensional projection, and (d) whitened transform for various values of the spectral decay exponent, α. (e) Eigenvalues of the latent covariance, Ω, for different decay rates, α. (f-h) Multi-task generalization error as a function of training samples, p, for (f) the latent variables themselves, (g) the random projection, and (h) the whitened transform.

2 Validation on Gaussian simulations

We validate our theory numerically on data drawn from a Gaussian model. Here, we find that our theory yields an excellent agreement with numerical simulations for a wide range of values (Methods). In these simulations, we sample the latents z_μ and neural responses x_μ from a multivariate Gaussian. We set the covariance matrix of the latents to have a spectrum that decays as a power law. Since the proof of our main theorem assumes that the spectra of the covariances decays slowly, this allows us to parametrically study violations of our main theorem's assumptions (Fig. 1a-e; SM). After sampling the latents, we form the neural responses either by taking a random high dimensional projection of the latent variables, or by applying a whitening transform to the latent variables (Fig. 1b-d; see Methods). We then calculate the empirical generalization error across a set of binary classification tasks which are formed by shattering the latent space as above.

As shown in Fig. 1f-h, our theory yields an excellent fit to numerical simulations. Importantly, the theoretical prediction holds all the way down to the few shot learning regime in which the number of training samples p is small, despite the theory being derived in the limit of large p. Furthermore, we find that the theory predicts the empirical generalization error well for a relatively small number of neurons n and latent dimensionality d, as well as latent variables whose covariance has an eigenspectrum that decays relatively quickly [4] (Methods). These results suggest that our theory can be applied to a wide range of datasets and is informative of the few shot learning regime.

3 Decomposition of generalization error into geometric terms

Having obtained a closed-form solution for generalization error in the thermodynamic limit, we now seek to rewrite this formula as a monotone function of interpretable geometric terms. We start by splitting Eq. (54) into a p-dependent and p-independent term:

$$\frac{1}{\pi}\tan^{-1}\left(\sqrt{\frac{\pi}{2p}\frac{\mathrm{Tr}(\Omega_n)\mathrm{Tr}(\Psi_n^2)\mathrm{Tr}(\Omega_n)}{(\mathrm{Tr}(\Phi_n\Phi_n^\top))^2}+\frac{\mathrm{Tr}(\Omega_n)\mathrm{Tr}(\Phi_n^\top\Psi_n\Phi_n)}{(\mathrm{Tr}(\Phi_n\Phi_n^\top))^2}-1}\right). \tag{55}$$

If we re-write the p-dependent term as

$$\frac{\pi}{2p}\left(\frac{\mathrm{Tr}(\Omega_n)\,\mathrm{Tr}(\Psi_n)}{\mathrm{Tr}(\Phi_n\Phi_n^\top)}\right)^2\cdot\frac{\mathrm{Tr}(\Psi_n^2)}{\mathrm{Tr}(\Psi_n)^2}, \tag{56}$$

we can notice the **participation ratio of the neural activity**

$$\mathrm{PR}(\Psi)=\frac{\mathrm{Tr}(\Psi_n)^2}{\mathrm{Tr}(\Psi_n^2)}, \tag{57}$$

which is a well-known measure of the dimensionality of neural activity. We collect the rest of the p-dependent term into our **correlation term**

$$c=\frac{\mathrm{Tr}(\Phi_n\Phi_n^\top)}{\mathrm{Tr}(\Omega_n)\,\mathrm{Tr}(\Psi_n)}, \tag{58}$$

and the generalization error now reduces to

$$\frac{1}{\pi}\tan^{-1}\left(\sqrt{\frac{\pi}{2pc^2\,\mathrm{PR}(\Psi)}+\frac{\mathrm{Tr}(\Omega_n)\mathrm{Tr}(\Phi_n^\top\Psi_n\Phi_n)}{(\mathrm{Tr}(\Phi_n\Phi_n^\top))^2}-1}\right). \tag{59}$$

To interpret the correlation term, note that the numerator $\mathrm{Tr}(\Phi_n\Phi_n^\top)$ can be expanded as

$$\mathrm{Tr}(\Phi_n\Phi_n^\top)=\sum_{i=1}^{d}\sum_{j=1}^{n}\mathbb{E}[z_i x_j]^2, \tag{60}$$

which is a sum-of-squares of the covariance between all pairs of latent variables z_i and all components x_j of neural activity. The denominator of c is just the total latent variance $\mathrm{Tr}(\Omega_n)$ times the total variance $\mathrm{Tr}(\Psi_n)$ of neural activity. The correlation term c can therefore be viewed as a generalization of the well-known Pearson correlation between two scalar variables to capture the total correlation strength between the d-dimensional latent variable z and the n-dimensional neural activity x. In particular, when $n, d = 1$, the total correlation c reduces to the square of the standard Pearson correlation between z and x.

Before moving on, we restrict ourselves to the case of diagonal latent covariance with

$$\Omega_n=\mathrm{diag}(\omega_1,\omega_2,\ldots,\omega_n).$$

Note that for *any* latent covariance, we can rotate the latent space to diagonalize Ω. Rotating the latent space does not affect our generalization error at all because we sample the task-defining vectors T from a rotationally-symmetric standard Gaussian distribution. The statements we make below about the latent variables $z_1, z_2, \ldots, z_d$ can more generally be interpreted as holding for the d *principal component directions* of the latent distribution when Ω_n is not diagonal, since these principal component directions will be rotated onto the d standard axes when we diagonalize Ω_n.

We call (the inverse of) the remaining p-independent term the **alignment term** a:

$$a = \frac{(\operatorname{Tr}(\Phi_n \Phi_n^\top))^2}{\operatorname{Tr}(\Omega_n) \operatorname{Tr}(\Phi_n^\top \Psi_n \Phi_n)}.$$

In the main text (and as described below), the alignment term is subdivided further, but we can also interpret a as a whole. We can expand the main term in the denominator as

$$\operatorname{Tr}(\Phi_n^\top \Psi_n \Phi_n) = \sum_{i=1}^{d} \phi_i^\top \Psi_n \phi_i = \sum_{i=1}^{d} \|\phi_i\|^2 \operatorname{Var}(\hat{\phi}_i \cdot x), \tag{61}$$

where ϕ_i is the ith column of Φ_n, and $\operatorname{Var}(\hat{\phi}_i \cdot x)$ is the variance of the total neural response x projected onto the direction of ϕ_i. Using the fact that the average neural activity, conditioned on a specific value of the latents is given by $\mathbb{E}[x|z] = \Phi\Omega^{-1}z$, we can see that for a diagonal Ω, the column $\hat{\phi}_i$ corresponds to the *coding direction of the latent variable* z_i. Additionally, the norm $\|\phi_i\|$ reflects the *coding strength of the latent variable* z_i. The numerator of a can be written as

$$(\operatorname{Tr}(\Phi_n \Phi_n^\top))^2 = \left(\sum_{i=1}^{d} \|\phi_i\|^2 \right), \tag{62}$$

and therefore depends only on the norms of the coding directions ϕ_i but not on their directions. Holding the norms of each ϕ_i fixed, we can see that the alignment term a prefers that each coding direction $\hat{\phi}_i$ be positioned along a direction of minimal response variance in the neural state space. In other words, the only variance along a direction $\hat{\phi}_i$ should be caused by variations in the corresponding latent variable. Intuitively, the alignment term a encourages arranging the coding directions of each latent variable in such a way that the signal for these variables do not interfere with one another and so that all latents are coded along directions with low noise. We make this notion more formal below.

Now let us subdivide the alignment term a as in the main text. We partition the total neural variance Ψ_n into stimulus-driven variance and stimulus-independent variance. Recall that in our setup, latents z_n and neural responses x_n are drawn from a joint Gaussian distribution

$$(x_n, z_n) \sim \mathcal{N}\left(0, \begin{pmatrix} \Omega_n & \Phi_n^\top \\ \Phi_n & \Psi_n \end{pmatrix}\right). \tag{63}$$

This is equivalent to a model in which we first sample $z_n \sim \mathcal{N}(0, \Omega_n)$ and then sample x_n as $x_n = \Phi_n \Omega_n^{-1} z_n + \varepsilon_n$, where ε_n is stimulus-independent Gaussian noise with a covariance given by

$$\Psi_n - \Phi_n \Omega_n^{-1} \Phi_n^\top, \tag{64}$$

which is the covariance of the neural responses conditional on the latents, $\mathrm{cov}(x_n|z_n)$. This suggests a partitioning of the total neural variance Ψ_n into stimulus-independent variance, $H_n := \Psi_n - \Phi_n\Omega_n^{-1}\Phi_n^\top$, and stimulus-driven variance, $\mathrm{cov}(x) - H_n = \Phi_n\Omega_n^{-1}\Phi_n^\top$.

We can now decompose the p-independent term of our generalization error formula as

$$\frac{\mathrm{Tr}(\Omega_n)\,\mathrm{Tr}(\Phi_n^\top\Psi_n\Phi_n)}{(\mathrm{Tr}(\Phi_n\Phi_n^\top))^2} = \frac{\mathrm{Tr}(\Omega_n)\,\mathrm{Tr}(\Phi_n^\top H_n\Phi_n)}{(\mathrm{Tr}(\Phi_n\Phi_n^\top))^2} + \frac{\mathrm{Tr}(\Omega_n)\,\mathrm{Tr}(\Phi_n^\top(\Phi_n\Omega_n^{-1}\Phi_n^\top)\Phi_n)}{(\mathrm{Tr}(\Phi_n\Phi_n^\top))^2}. \tag{65}$$

We call the inverse of the first term **signal-noise factorization** s:

$$\frac{1}{s} = \frac{\mathrm{Tr}(\Omega_n)\,\mathrm{Tr}(\Phi_n^\top H_n\Phi_n)}{(\mathrm{Tr}(\Phi_n\Phi_n^\top))^2}. \tag{66}$$

This interpretation can be understood by noting that

$$\mathrm{Tr}(\Phi_n^\top H_n\Phi_n) = \sum_{i=1}^{d}\phi_i^\top H_n\phi_i = \sum_{i=1}^{d}\|\phi_i\|^2\cdot\hat{\phi}_i^\top H_n\hat{\phi}_i. \tag{67}$$

We can see that $\hat{\phi}_i^\top H\hat{\phi}_i$ gives the projection of stimulus-independent noise along the coding direction $\hat{\phi}_i$ of the ith latent variable, so $\mathrm{Tr}(\Phi_n^\top H\Phi_n)$ measures the amount of stimulus-independent noise corrupting the signal directions for each latent variable. In particular, note that s is maximized when there is no stimulus-independent noise in the neural dimensions used for coding the latent variables.

Finally, we call the inverse of the second part of the p-independent term the **signal-signal factorization**:

$$\frac{1}{f} = \frac{\mathrm{Tr}(\Omega_n)\,\mathrm{Tr}(\Phi_n^\top(\Phi_n\Omega_n^{-1}\Phi_n^\top)\Phi_n)}{(\mathrm{Tr}(\Phi_n\Phi_n^\top))^2}. \tag{68}$$

To understand this term, recall our assumption that

$$\Omega_n = \mathrm{diag}(\omega_1,\ldots,\omega_d), \tag{69}$$

which can be accomplished by making an orthogonal change of variables. Now observe that

$$\mathrm{Tr}(\Phi_n^\top\Phi_n\Omega_n^{-1}\Phi_n^\top\Phi_n) = \sum_{i=1}^{d}\sum_{j=1}^{d}\frac{1}{\omega_i}\langle\phi_i,\phi_j\rangle^2. \tag{70}$$

The numerator of $1/f$ is a weighted sum of the dot products between *all pairs* of coding vectors ϕ_i and ϕ_j for latents z_i and z_j. By contrast, the denominator $(\mathrm{Tr}(\Phi_n\Phi_n^\top))^2$ can be expanded to

$$(\mathrm{Tr}(\Phi_n\Phi_n^\top))^2 = \left(\sum_{i=1}^{d}\langle\phi_i,\phi_i\rangle\right)^2, \tag{71}$$

which only captures the signal strengths $\|\phi_i\|$ without regard for the angles between pairs of distinct signal directions. With the norms $\|\phi_i\|$ fixed, f is maximized by making all coding

directions orthogonal (and more generally for non-diagonal Ω, by mapping the PCs of the latents to orthogonal directions). That is, f is maximized by a factorized code.

The interpretation of our factorization term f is somewhat complicated by its dependence on the latent covariance matrix Ω, especially when Ω can change between conditions (e.g., the distribution of the rats' positions can change between sessions in our analysis of CA1 and PFC spatial codes) or when latents are strongly correlated (e.g., the positions of different marker locations in the pose estimation data are highly correlated). We therefore compare the signal-signal factorization to a simplified measure that removes the dependence on Ω completely:

$$\frac{1}{f_{\text{simplified}}} = \frac{\text{Tr}(\Phi_n^\top \Phi_n \Phi_n^\top \Phi_n)}{(\text{Tr}(\Phi_n \Phi_n^\top))^2}, \tag{72}$$

Letting θ_{ij} denote the angle between the coding directions $\hat{\phi}_i$ and $\hat{\phi}_j$ and defining weights $w_{ij} = \|\phi_i\|^2 \|\phi_j\|^2$, we can rewrite

$$\frac{1}{f_{\text{simplified}}} = \frac{\sum_{i,j=1}^{D} w_{ij} (\cos\theta_{ij})^2}{\sum_{i,j=1}^{D} w_{ij}} \tag{73}$$

so that $1/f_{\text{simplified}}$ is just a weighted average of the squared cosine angle between all pairs of coding directions and is manifestly optimized by a factorized code with $\cos\theta_{ij} = 0$ for $i \neq j$. The weighting by the signal norms $\|\phi_i\|$ is unavoidable. For example, if one coding direction ϕ_i has norm very near zero (i.e., latent z_i is not decodable at all), then the angle it makes with other coding directions is irrelevant to generalization error and should not enter into f, since our measures capture the geometry *relevant for generalization error*.

We demonstrate numerically that practically all of the variation we observe in f across all of our analyses is captured by $f_{\text{simplified}}$ and is therefore independent of any changes in the latent covariance Ω (SM Fig. 2).

4 Optimal geometry

Here we derive our formula for the spectrum of the optimal neural representation and show that it is disentangled. The argument is not fully rigorous, but it matches numerical results very well.

Let us begin by showing that directions in the latent space with more variance are, on average, more informative of the task labels than those with low variance. To do this, consider a latent variable z_i and a label for a given task: $y = \text{sgn}(z \cdot r)$, where as above r denotes the normal vector of the hyperplane defining the task and z is the vector of all latent variables. The correlation between this latent variable and the labels for this task is given by, $\rho := \mathbb{E}_z[z_i \text{sgn}(z \cdot r)]/\sqrt{\mathbb{E}_z[z_i^2]\mathbb{E}_z \text{sgn}(z \cdot r)^2}$. Thus, we can quantify how informative a latent direction is for the labels across all tasks by calculating $\mathbb{E}_r|\rho|$, where the absolute value is taken to break the symmetry between positive and negative ρ values as we average over r. Using Eq. (7), together with the concentration of $r \cdot \Omega r$ about its mean value, we find that for large d:

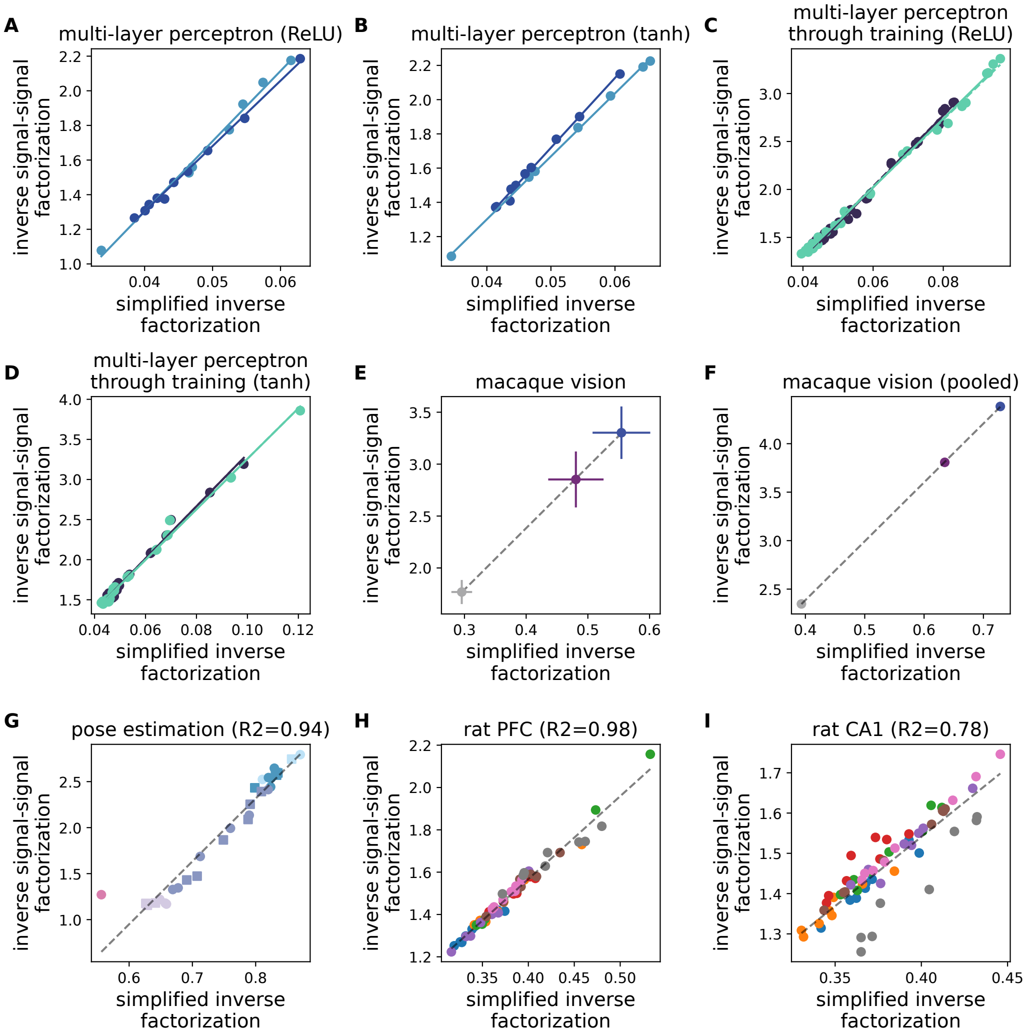

Figure 2: Almost all variation in our factorization term is driven by changing angles between coding directions and not by changes in the latent covariance Ω. We compare the reciprocal of signal-signal factorization to a simplified formula that can be written as a weighted average of squared cosines between pairs of coding directions and does not depend on the latent covariance Ω (see Eq. (73)). The strong linear relationship between $1/f$ and the simplified formula shows that we can effectively consider f to not depend on the latent covariance Ω across all the experiments presented in this work. The legend for each panel is not reprinted for brevity but matches the experimental figures. (a) Random and trained ReLU networks (Fig. 3). (b) Random and trained tanh networks (SM Fig. 5). (c) ReLU networks across training (SM Fig. 4). (d) Tanh networks across training (SM Fig. 6). (e) Macaque ventral stream (Fig. 5, error bars denote SEM and points the mean across categories). (f) Macaque ventral stream (pooled categories, SM Fig. 11). (g) Deep pose estimation network (Fig. 4) (h-i) Rat CA1 and PFC (Fig. 7)

$$\mathbb{E}_r|\rho| = \frac{\sqrt{2}}{\sqrt{\pi\Omega_{ii}\mathrm{Tr}(\Omega)}}\mathbb{E}_r|(\Omega r)_i| = \frac{2\sqrt{(\Omega^\top\Omega)_{ii}}}{\pi\sqrt{\Omega_{ii}\mathrm{Tr}(\Omega)}}. \tag{74}$$

If Ω is diagonal (or, equivalently, if we consider the correlation of the label with a principle component of Ω), then this is merely proportional to $\Omega_{ii}^{1/2}/\sqrt{\mathrm{Tr}(\Omega)}$–i.e. the standard deviation of the latent, normalized by the square root of the total variance across all latent variables, $\mathrm{Tr}(\Omega)$. More generally, this equation shows that latents with large variance are more informative of task labels.

With these considerations in place, our goal is to calculate

$$\arg\min_{\Psi,\Phi} \frac{1}{\pi}\tan^{-1}\left(\frac{\sqrt{\mathrm{Tr}(\Omega)\left[\frac{\pi}{p}\mathrm{Tr}(\Psi^2)\mathrm{Tr}(\Omega) + 2\left(\mathrm{Tr}(\Phi^\top\Psi\Phi) - \frac{(\mathrm{Tr}[\Phi\Phi^\top])^2}{\mathrm{Tr}(\Omega)}\right)\right]}}{\sqrt{2}\mathrm{Tr}(\Phi\Phi^\top)}\right), \tag{75}$$

subject to

$$\begin{pmatrix} \Psi & \Phi \\ \Phi^\top & \Omega \end{pmatrix} \succ 0. \tag{76}$$

For a positive matrix Ω, this condition is equivalent to

$$\Psi \succ 0, \quad \Psi - \Phi\Omega^{-1}\Phi^\top \succ 0. \tag{77}$$

Rewriting the argument of the $\tan^{-1}$ function, we can see that the objective may be written as

$$\arg\min_{\Psi,\Phi} \frac{\frac{\pi}{p}\,\mathrm{Tr}(\Psi^2)\,\mathrm{Tr}(\Omega) + 2\,\mathrm{Tr}(\Phi^\top\Psi\Phi)}{\mathrm{Tr}(\Phi\Phi^\top)^2}. \tag{78}$$

Our approach is to first minimize Eq. (78) holding $\mathrm{Tr}(\Phi\Phi^\top) = \gamma$ fixed and show that the objective is ultimately invariant to the choice of γ. The optimization can be done by introducing the Lagrangian

$$L(\Psi,\Phi,\rho,\zeta) = c\mathrm{Tr}(\Psi^2) + 2\mathrm{Tr}(\Phi^\top\Psi\Phi) + \zeta(\gamma - \mathrm{Tr}(\Phi\Phi^\top)) \tag{79}$$

$$- \int d^n x \rho(x)\mathrm{Tr}(xx^\top(\Psi - \Phi\Omega^{-1}\Phi^\top)), \tag{80}$$

where the $\rho(x)$ are KKT multipliers enforcing the positive definite constraint, ζ is a Lagrange multiplier, and $c := \frac{\pi}{p}\mathrm{Tr}(\Omega)$. Note that we do not explicitly enforce the positivity constraint on Ψ, as we find that this is unnecessary. The KKT equations are

$$\partial_\Psi L = 2c\Psi + 2\Phi\Phi^\top - \langle xx^\top \rangle_\rho = 0, \tag{81}$$

$$\partial_\Phi L = 4\Psi\Phi + 2\langle xx^\top \rangle \Phi\Omega^{-1} - 2\zeta\Phi = 0, \tag{82}$$

$$\delta_\rho L = \mathrm{Tr}(xx^\top(\Psi - \Phi\Omega^{-1}\Phi^\top)) \geq 0, \tag{83}$$

$$\rho(x)\mathrm{Tr}(xx^\top(\Psi - \Phi\Omega^{-1}\Phi^\top)) = 0, \tag{84}$$

$$\mathrm{Tr}(\Phi\Phi^\top) = \gamma. \tag{85}$$

We try for a solution with $\Psi = \Phi\Omega^{-1}\Phi^\top$. Since the variance of x conditional on z is given by $\Psi - \Phi\Omega^{-1}\Phi^\top$, this is equivalent to looking for solutions in which the neural code has no signal-unrelated noise. Then Eq. (81) gives $\langle xx^\top \rangle_\rho = 2\Phi(c\Omega^{-1} + I)\Phi^\top$. Using these two formulae, Eq. (82) gives the condition

$$\Phi(4\Omega^{-1}\Phi^\top\Phi + 4(c\Omega^{-1} + I)\Phi^\top\Phi\Omega^{-1} - 2\zeta I) = 0. \tag{86}$$

This suggests looking for a Φ whose right singular vectors are aligned with those of Ω. The singular values of Φ are then given by:

$$\phi_i^2 = \frac{\gamma}{\sum_i \frac{\omega_i^2}{2\omega_i + \frac{\pi}{p}\mathrm{Tr}(\Omega)}} \frac{\omega_i^2}{2\omega_i + \frac{\pi}{p}\mathrm{Tr}(\Omega)}. \tag{87}$$

From the assumption that $\Psi = \Phi^\top\Omega^{-1}\Phi$, we can see that the eigenvectors of Ψ and the left singular vectors of Φ can be chosen however we want, so long as they are the same. The eigenvalues of Ψ are then simply

$$\psi_i = \frac{\gamma}{\sum_i \frac{\omega_i^2}{2\omega_i + \frac{\pi}{p}\mathrm{Tr}(\Omega)}} \frac{\omega_i}{2\omega_i + \frac{\pi}{p}\mathrm{Tr}(\Omega)}. \tag{88}$$

Plugging this solution into the objective, we indeed find that γ drops out of the picture entirely. Since we are free to choose γ, we obtain that the optimal representation satisfies the following properties: (1) The eigenvectors of Ψ are the left singular vectors of Φ and (2) The eigenvectors of Ω are the right singular vectors of Φ.

We now show that the optimal representation described above is disentangled. More precisely, we show that the principal components of z directly map onto the principal components of x. If we let o be the eigenvector of Ψ corresponding to the neural direction and u the eigenvector of Ω corresponding to the latent direction, we can see that the value of

$$\mathbb{E}[\langle o, x\rangle\langle u, z\rangle] = u^\top\Phi o. \tag{89}$$

is either 0 or equal to a singular value of Φ. It is then easy to check that $\mathrm{corr}(\langle o, x\rangle, \langle u, z\rangle)$ is similarly either 0 or 1. Thus the principal components of the neurons and latents stand in a one to one relationship to one another. When the individual latent variables are uncorrelated

with one another (i.e. when Ω is diagonal), this implies that distinct latent variables are represented along mutually orthogonal directions.

Finally, the eigen/singular-values of the matrices are given by

$$\phi_i^2 \propto \frac{\omega_i^2}{2p\omega_i + \pi \mathrm{Tr}(\Omega)}, \tag{90}$$

$$\psi_i \propto \frac{\omega_i}{2p\omega_i + \pi \mathrm{Tr}(\Omega)}. \tag{91}$$

This gives the optimal representation's spectrum. As stated in the main text, we can see that as p grows relative to d, the spectrum becomes increasingly flat, indicating that more variance in the neural state space is being allocated to the less informative directions in the latent space.

5 Comparison of all analyses to SVC

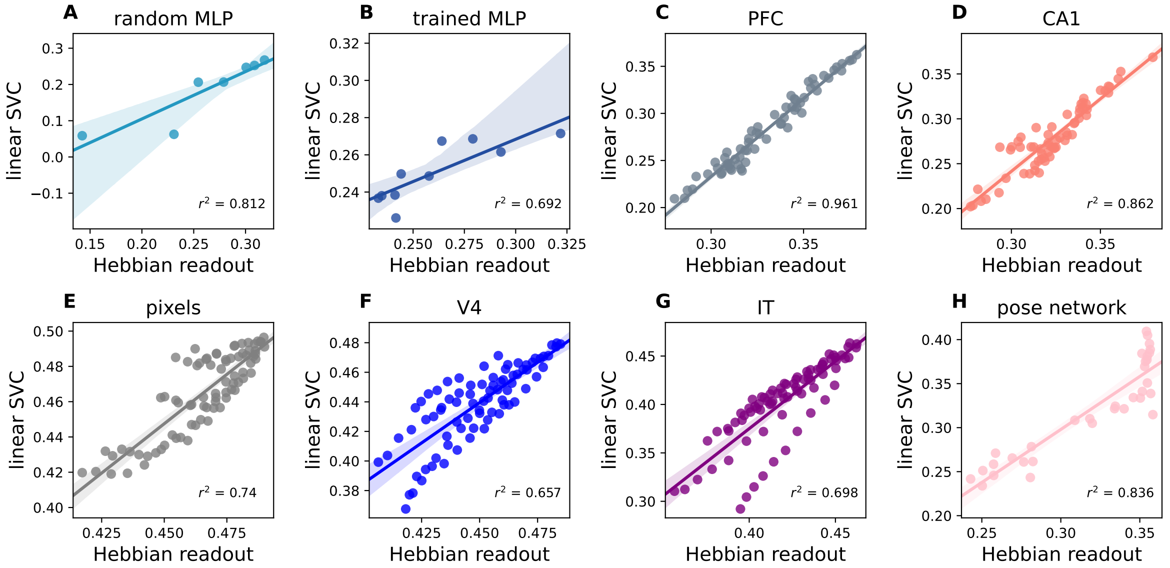

Figure 3: Comparison of the theoretically calculated Hebbian readout and a linear support vector classifier (SVC) on the multi-task learning problems analyzed in the main text. We fit a linear SVC using the default parameters in scikitlearn and average over train/test splits, as well as distinct shatterings of the latent space. Across all analyses reported in the main text, we find a strong linear association between the performance of the two readouts, suggesting that the geometric statistics we derived using Hebbian readouts are also informative of the performance of non-Hebbian readouts. (a-b) Random and trained MLP analyses across all layers for fixed p. (c-d) PFC and CA1 analyses across all rats and sessions. (e-g) Pixels, V4, and IT analyses for all values of p reported in the main text. (h) DeepLabCut results using the same values of p reported in the main text.

6 Additional MLP analyses

Here, we present results regarding the evolution of the geometric terms through training. Additionally, we redo the MLP analyses using a tanh non-linearity in the random and trained networks, and we consider a trained network with a similar fanout structure to the random network.

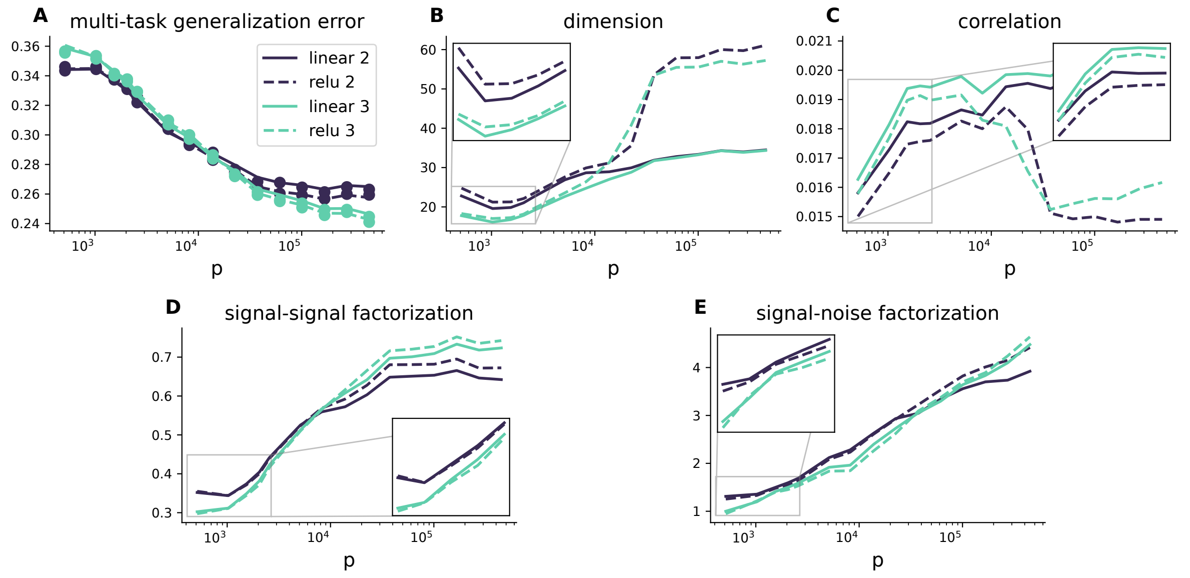

Figure 4: Evolution of generalization error and representational geometry through training. Note that p here denotes the number of stochastic gradient descent (SGD) training steps. (a) Generalization error of the Hebb rule applied to four different layers on a previously unseen set of tasks. We examine layers from the early stages of the MLP (black) as well as late stages (green) for both linear (solid line) and relu (dashed line) layers. We can see that the error decreases across all layers with the number of training samples, p. (b-e) Evolution of the geometry over the course of learning. Insets show the geometric terms during the first five steps of SGD. Just as in the rat analyses of Sec. 3.6, we find that nearly all geometric terms improve uniformly at the beginning of learning. Moreover, we find a similar fall-then-rise trend in the total dimension in these early stages. Late in training, we find that the dimension increases, and we see a non-monotonic trend in the correlation, whereby it begins to decrease late in training in the relu layers. Both factorization terms uniformly increase through training across the network, though the SSF begins to plateau at the end of training. The geometric trends we observe late in training mirror the effect of increasing the number of training samples on the optimal neural code.

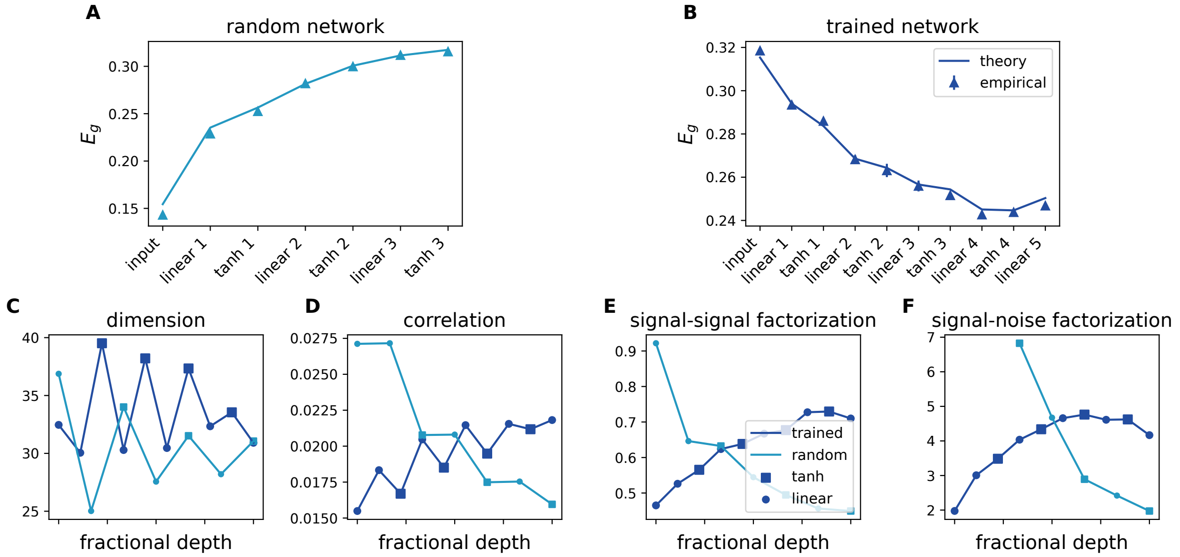

Figure 5: Generalization error and geometry of the tanh MLPs. (a-b) Generalization error for the random and trained network. (c-f) Layer-wise geometry of the networks. We find similar trends as with the relu non-linearity.

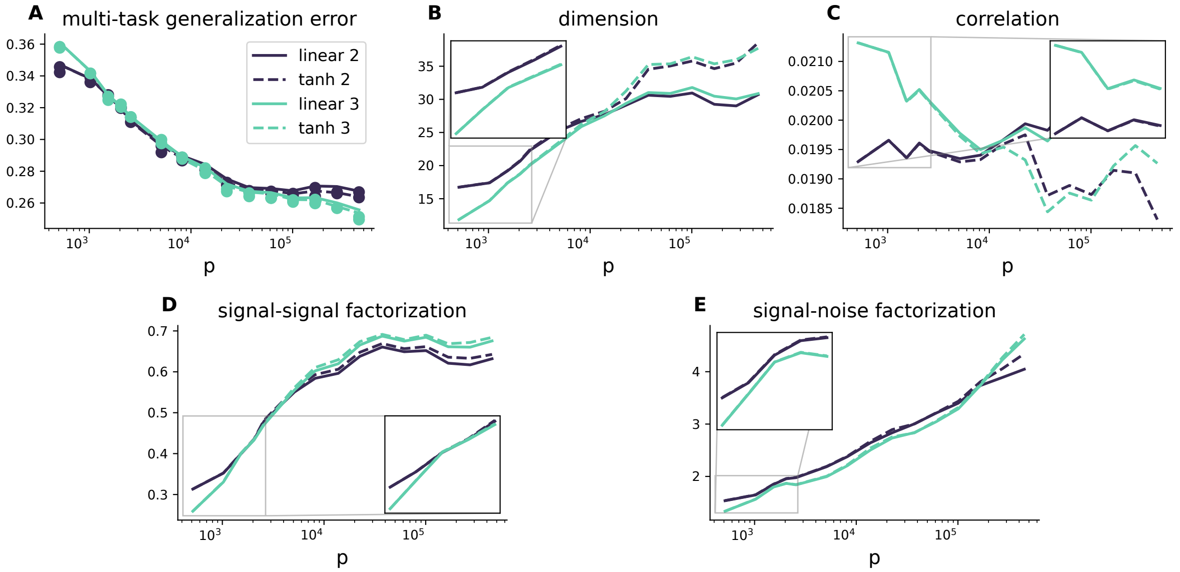

Figure 6: Dynamics of generalization error (a) and geometry (b-e) through training using networks with a tanh non-linearity. Again, we show 2 early and 2 late layers. At each of the early/late stages, we show a linear and tanh layer. Inset denotes geometric quantities over the first five steps of SGD on a linear scale. We see that with the exception of the correlation, the geometric terms uniformly improve early in learning, with non-monotonic effects beginning in the later stages of learning.

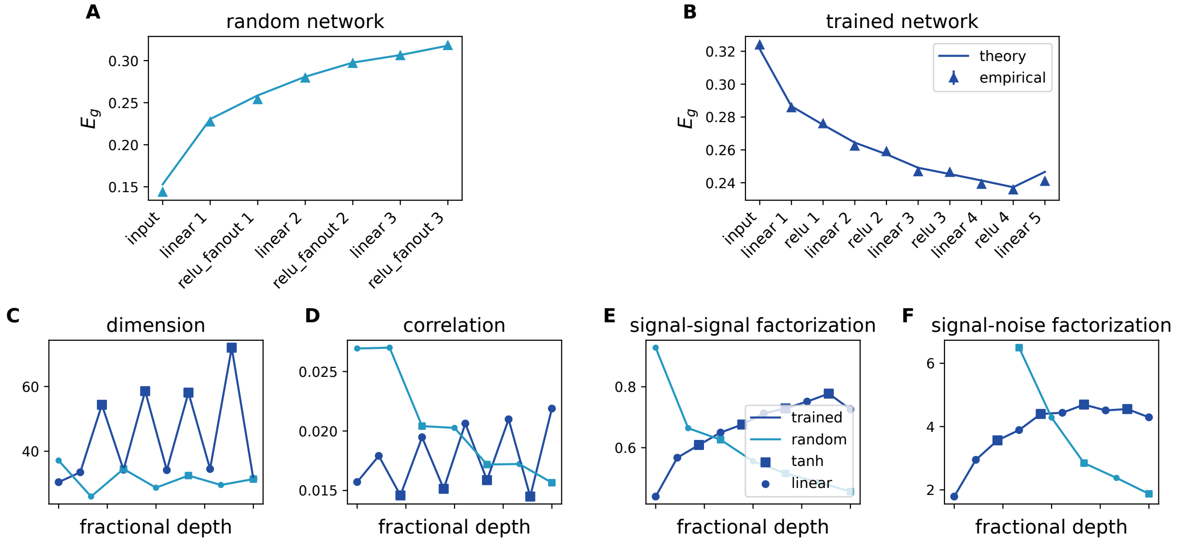

Figure 7: Generalization error and geometry of the relu MLPs in which the trained network has a similar expansionary structure as the random network. Specifically, the trained network has a layer structure in which each layer is 1.5 times the size of the previous one. We find similar trends as those reported in the main text. (a-b) Generalization error in both networks. (c-f) Geometry across layers.

7 Additional macaque analyses

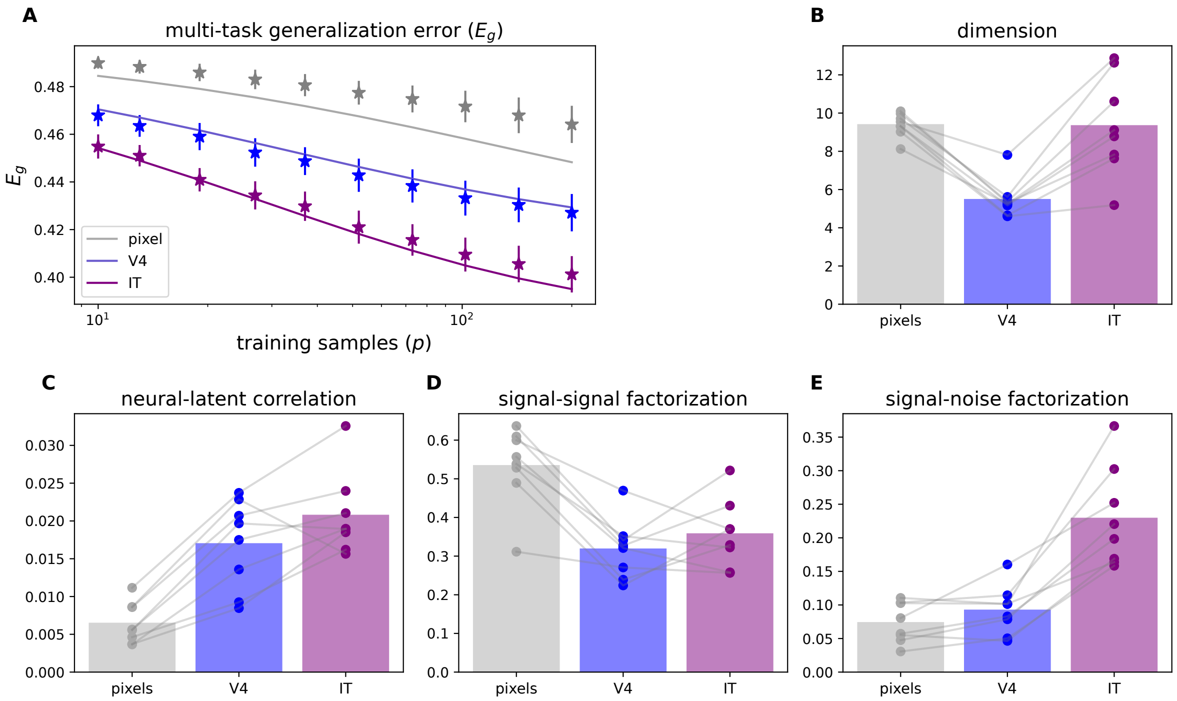

Figure 8: Geometry and generalization error using the same number of units across regions [2]. Here, we repeat the analysis presented in the main text, only we project the pixels and IT data down to 88 dimensions using Gaussian random projection. (a) Generalization error for the three representations. (b-e) Geometric terms. We find qualitatively similar trends to those presented in the main text.

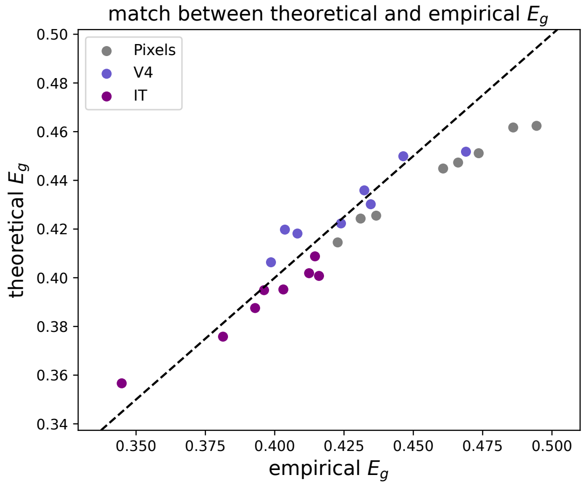

Figure 9: Match between theoretical and empirical generalization error across each of the 8 individual categories. In the main text, we presented the theoretical and empirical generalization errors averaged over individual object categories. Here we show that the theory predicts the empirical generalization error well across individual object categories.

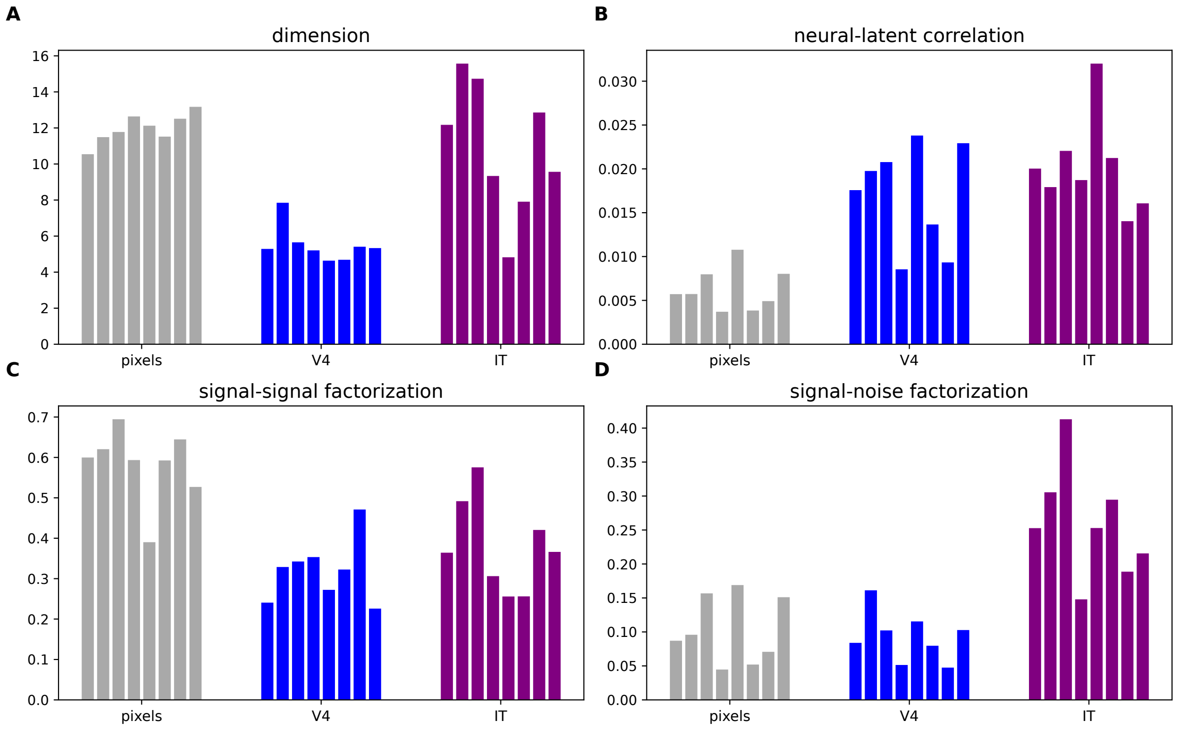

Figure 10: Geometry across individual object categories. Here, we present the distribution of geometric terms, calculated on subsets of the data corresponding to each of the 8 object categories.

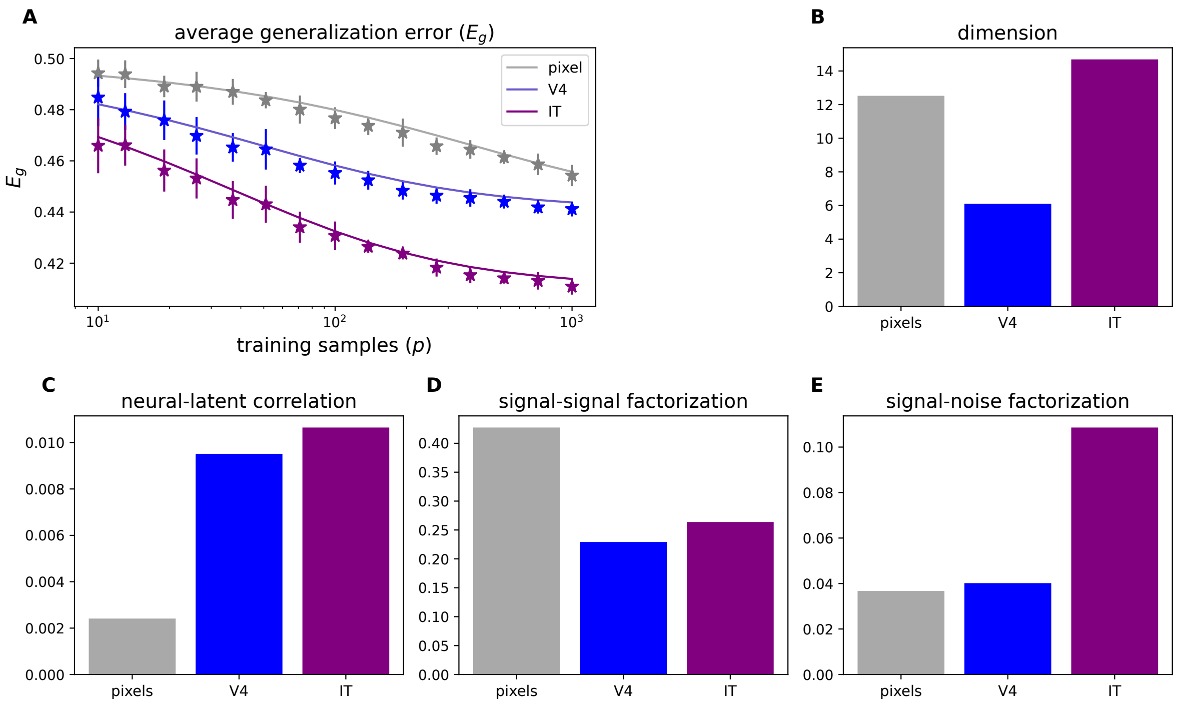

Figure 11: Generalization error and geometry of the pooled monkey data. Here, we pool data from all categories together, rather than considering data subsets corresponding to stimuli coming from the same object category as done in the main text. We can see that the trends are largely the same and that the theory predicts the empirical error. (a) Theoretical and empirical generalization error. (b-e) Geometry of the pooled data.

Measure	Group 1	Group 2	t	p	p (adj.)
dimension	pix	V4	13	3.8e-06	4.6e-05
dimension	pix	IT	0.791	0.455	0.496
dimension	V4	IT	-5.17	0.00129	0.00194
corr.	pix	V4	-7.49	1.4e-04	4.2e-04
corr.	pix	IT	-9.23	3.6e-05	1.8e-04
corr.	V4	IT	-1.6	0.154	0.205
ssf	pix	V4	8.89	4.6e-05	1.8e-04
ssf	pix	IT	7.13	1.9e-04	4.5e-04
ssf	V4	IT	-1.44	0.192	0.231
snf	pix	V4	0.682	0.517	0.517
snf	pix	IT	-5.94	5.7e-04	9.8e-04
snf	V4	IT	-6.84	2.4e-04	4.9e-04

Table 1: Paired sample two-sided t-test results presented in Fig. 5. All p-values were adjusted using a Benjamini-Hochberg method.

8 Additional rat analyses

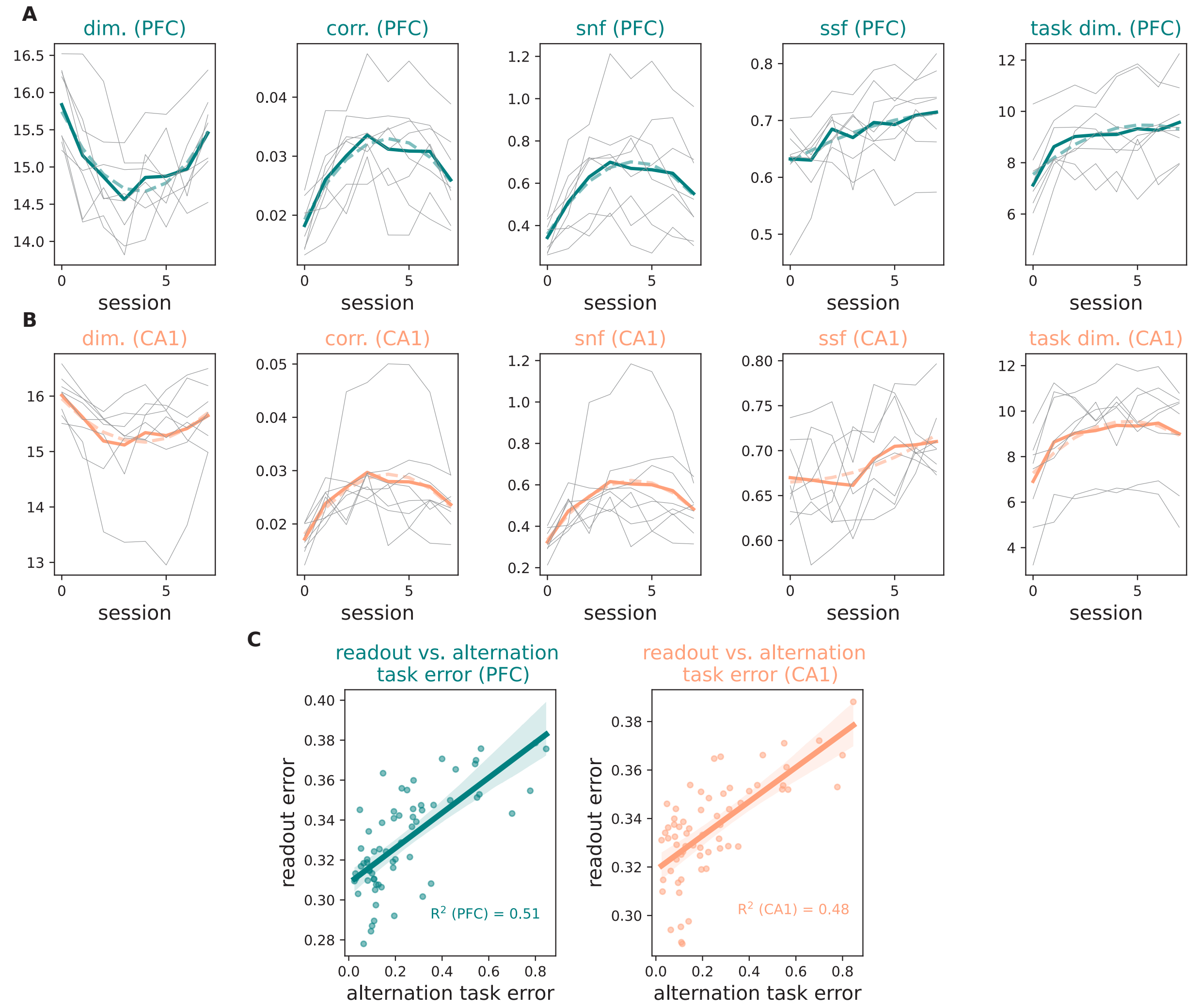

Figure 12: (A-B) Example quadratic model fits (dashed lines) for PFC (A) and CA1 (B). Geometric measures for individual rats ($N = 8$) shown in light grey and population averages in solid colored lines. (To test the effect of the outlier rat in the CA1 data, we reran all statistical tests after excluding this rat's CA1 data. Besides the linear model's CA1 SSF term, no other term ceased to be statistically significant at $\alpha = 0.01$ after performing this exclusion.) (C) Relationship between single session error rates and theoretically calculated E_g.

Area	Model	Measure	γ	p_γ	p_γ (adj.)	η	p_η	p_η (adj.)
PFC	Quad.	task dim.	0.716	7.0e-06	9.4e-06	-0.0662	0.00247	0.00297
PFC	Quad.	snf	0.164	2.8e-14	2.3e-13	-0.0197	3.1e-11	1.2e-10
PFC	Quad.	ssf	0.0204	0.0267	0.0305	-0.00117	0.352	0.375
PFC	Quad.	corr.	0.00677	1.3e-17	3.1e-16	-8.3e-04	1.8e-14	2.1e-13
PFC	Quad.	dim.	-0.574	6.9e-14	4.2e-13	0.0765	3.7e-13	1.8e-12
PFC	Linear	ssf	0.0122	1.4e-06	2.2e-06			
PFC	Linear	task dim.	0.252	7.6e-08	1.8e-07			
CA1	Quad.	task dim.	0.997	8.8e-11	3.0e-10	-0.108	2.8e-07	5.7e-07
CA1	Quad.	snf	0.139	2.4e-08	7.3e-08	-0.0169	8.4e-07	1.3e-06
CA1	Quad.	corr.	0.00557	2.9e-08	7.7e-08	-6.9e-04	5.7e-07	1.1e-06
CA1	Quad.	ssf	5.3e-04	0.947	0.947	1.0e-03	0.36	0.375
CA1	Quad.	dim.	-0.409	1.8e-07	4.0e-07	0.0532	7.9e-07	1.3e-06
CA1	Linear	ssf	0.0075	5.5e-04	7.0e-04			
CA1	Linear	task dim.	0.238	3.1e-06	4.3e-06			

Table 2: Parameter estimates and associated p-values for the quadratic regression models (Methods). Here, we fit mixed-effects models of geometric terms. A given geometric measure y for rat i in session t was modeled as $y_{it} = \beta_i + \gamma t + \eta t^2$. We additionally fit a linear model to the SSF term of the form $y_{it} = \beta_i + \gamma t$. All p-values were adjusted using a Benjamini-Hochberg method.

References

[1] Radoslaw Adamczak. “A note on the Hanson-Wright inequality for random vectors with dependencies”. In: (2015).

[2] Najib J Majaj et al. “Simple learned weighted sums of inferior temporal neuronal firing rates accurately predict human core object recognition performance”. In: *Journal of Neuroscience* 35.39 (2015), pp. 13402–13418.

[3] Martin Raič. “A multivariate Berry–Esseen theorem with explicit constants”. In: *Bernoulli* 25.4A (2019), pp. 2824–2853. DOI: `10.3150/18-BEJ1072`. URL: `https://doi.org/10.3150/18-BEJ1072`.

[4] Carsen Stringer et al. “High-dimensional geometry of population responses in visual cortex”. In: *Nature* 571.7765 (2019), pp. 361–365.

[5] Roman Vershynin. *High-dimensional probability: An introduction with applications in data science*. Vol. 47. Cambridge university press, 2018.
